# Gentiopicroside improves non-alcoholic steatohepatitis by activating PPARα and suppressing HIF1

**DOI:** 10.3389/fphar.2024.1335814

**Published:** 2024-03-07

**Authors:** Chaoyuan Huang, Qiuhong Yong, Yihui Lu, Lu Wang, Yiyuan Zheng, Lina Zhao, Peiwu Li, Chong Peng, Wei Jia, Fengbin Liu

**Affiliations:** ^1^ The Second Affiliated Hospital of Guangzhou University of Chinese Medicine, Guangzhou, China; ^2^ The First Clinical Medical School, Guangzhou University of Chinese Medicine, Guangzhou, China; ^3^ Lingnan Medical Research Center, Guangzhou University of Chinese Medicine, Guangzhou, China; ^4^ School of Chinese Medicine, Hong Kong Baptist University, Kowloon Tong, Hong Kong, China; ^5^ Department of Gastroenterology, Shanghai Municipal Hospital of Traditional Chinese Medicine, Shanghai University of Traditional Chinese Medicine, Shanghai, China; ^6^ Department of Hepatobiliary of The First Affiliated Hospital of Guangzhou University of Chinese Medicine, State Key Laboratory of Traditional Chinese Medicine Syndrome, Guangzhou, China; ^7^ Shanghai Key Laboratory of Diabetes Mellitus and Center for Translational Medicine, Shanghai Jiao Tong University Affiliated Sixth People’s Hospital, Shanghai, China; ^8^ Baiyun Hospital of The First Affiliated Hospital of Guangzhou University of Chinese Medicine, Guangzhou, China; ^9^ Lingnan Institute of Spleen and Stomach Diseases, The First Affiliated Hospital of Guangzhou University of Chinese Medicine, Guangzhou, China

**Keywords:** gentiopicroside, non-alcoholic steatohepatitis, metabolomics, fatty acid oxidation, peroxisome proliferator-activated receptor α, oxidative stress, hypoxia-inducible factor-1 α

## Abstract

Gentiopicroside (GPS) is a highly water-soluble small-molecule drug and the main bioactive secoiridoid glycoside of Gentiana scabra that has been shown to have hepatoprotective effects against non-alcoholic steatohepatitis (NASH), a form of non-alcoholic fatty liver disease (NAFLD) that can progress to cirrhosis and hepatocellular carcinoma. However, the effects of GPS on NASH and the underlying mechanisms remain obscure. Firstly, a high-fat, high-cholesterol (HFHC) diet and a high-sugar solution containing d-fructose and d-glucose were used to establish a non-alcoholic steatohepatitis (NASH) mice model. Secondly, we confirmed GPS supplementation improve metabolic abnormalities and reduce inflammation in NASH mice induced by HFHC and high-sugar solution. Then we used metabolomics to investigate the mechanisms of GPS in NASH mice. Metabolomics analysis showed GPS may work through the Peroxisome Proliferator-Activated Receptor (PPAR) signaling pathway and glycine, serine, and threonine metabolism. Functional metabolites restored by GPS included serine, glycine, eicosapentaenoic acid (EPA), and docosahexaenoic acid (DHA). Western blot and qRT-PCR analysis confirmed GPS improve NASH by regulating PPARα and Hypoxia-Inducible Factor-1α (HIF-1α) signaling pathways. *In vitro*, studies further demonstrated EPA and DHA enhance fatty acid oxidation through the PPARα pathway, while serine and glycine inhibit oxidative stress through the HIF-1α pathway in palmitic acid-stimulated HepG2 cells. Our results suggest GPS’s anti-inflammatory and anti-steatosis effects in NASH progression are related to the suppression of HIF-1α through the restoration of L-serine and glycine and the activation of PPARα through increased EPA and DHA.

## 1 Introduction

Non-alcoholic fatty liver disease (NAFLD) is a common condition that is characterized by excess fat accumulation in the liver (Ng et al., 2022). According to epidemiological studies, approximately one-third of the global population is affected by NAFLD (Le et al., 2019). Recently, some experts have proposed renaming NAFLD to metabolic dysfunction-associated steatotic liver disease (MASLD) to highlight the broader role of metabolic dysfunction in the disease (Hutchison et al., 2023; Rinella et al., 2023). NAFLD is divided into two categories: non-alcoholic fatty liver (NAFL) and non-alcoholic steatohepatitis (NASH). NAFL is generally considered to be benign and non-progressive, while NASH is the inflammatory form of the disease which may progress to cirrhosis and hepatocellular carcinoma (HCC). Some pharmaceutical treatments for NAFLD, such as lipid-lowering agents and insulin sensitizers, may carry a risk of adverse effects (Sharma et al., 2020).

Traditional Chinese medicine (TCM) has a long history of use in China and other parts of Asia. Gentiana scabra (GS), a commonly used herb in Tibet, is a type of gentian flower that has been shown to have anti-inflammatory and antioxidant properties (Choi et al., 2019). Our previous research has found that GS may be an effective option for the prevention and treatment of NAFLD (Zheng et al., 2021a). Gentiopicroside (GPS), as the main bioactive secoiridoid glycoside of GS, has been shown to have hepatoprotective effects, which are thought to be due to its ability to mitigate oxidative stress, decrease lipid synthesis, increase glucose utilization, attenuate mitochondrial dysfunction, and inhibit inflammation (Li et al., 2018; Cheng et al., 2019; Jin et al., 2020; Zhang et al., 2021). GPS, as a highly water-soluble compound, has the potential to become a small-molecule drug. However, the exact mechanisms by which GPS exerts these effects are not fully understood. Therefore, we propose that GPS may also be an effective treatment for NASH and further research is needed to clarify its pharmacological mechanisms for better dissemination.

Metabolomics is a field of study that uses advanced analytical chemistry techniques and statistical methods to comprehensively characterize the metabolites in a biological system (German et al., 2005; Kapoore et al., 2016). The metabolome is the complete set of all metabolites present in a system, and changes in the metabolome can provide insight into disease etiology and progression (Bujak et al., 2015; Kapoore et al., 2016; Wishart, 2019). Metabolites are the products of various physiological or pathophysiological processes, and they play important roles in many physiological functions and pathological processes (Chin et al., 2014). Evidence suggests that changes in metabolites can not only result from disturbances at the gene or protein level but also act as signaling molecules to intervene in pathophysiological states (Chin et al., 2014). Therefore, metabolomics is a valuable tool for investigating the potential effects of identified differential metabolites (Yan and Xu, 2018). There are two main approaches to metabolomics: targeted and untargeted. To fully capture the diversity of metabolites, it is often necessary to use a combination of approaches (Kapoore et al., 2016).

In this study, we used a combination of untargeted and targeted metabolomics to investigate the mechanisms of GPS in mice with NASH induced by a high-fat-high-cholesterol diets (HFHC) and the high-sugar solution including d-fructose and d-glucose. HFHC diet and high-sugar solution were considered as a reliable method to induce NASH and NASH-related liver cancer spontaneously and sequentially (Tsuchida et al., 2018; Zhang et al., 2021). Our results showed that GPS had multiple metabolic benefits. Using metabolomics approaches, we identified dozens of differential metabolites that were significantly altered in the liver tissues of either HFHC- or GPS-treated mice. Further pathway enrichment analysis revealed that the Peroxisome Proliferator-Activated Receptor (PPAR) signaling pathway, one of the crucial pathways in maintaining lipid homeostasis, was activated by GPS. *In vitro*, experiments also showed that certain amino acids and polyunsaturated fatty acids were able to inhibit oxidative stress and reduce lipid accumulation.

## 2 Materials and methods

### 2.1 Animal experimentation

Male C57BL/6 mice used in the experiments were 5 weeks old when they were purchased from Guangdong Medical Laboratory Animal Center. All mice were reared in a regulated barrier system facility at 23°C± 3°C with 55% ± 15% relative humidity and randomized equally into three groups after acclimation. Each group contains 8 mice. All the animal experiment protocols were approved by the Animal Care Welfare Committee of The First Affiliated Hospital of Guangzhou University of Chinese Medicine (Guangzhou, China) under a project license (TCMF1-2020028).

The mice in the control group were treated with a chow diet (CD, Guangdong Medical Laboratory Animal Center, Guangzhou, China). Other mice were fed with high-fat-high-cholesterol diets (HFHC, A-M07-D, Research Diets, Guangdong Medical Laboratory Animal Center, Guangzhou, China) and a high-sugar solution (23.1 g/L d-fructose (D809612, Macklin, Shanghai, China) and 18.9 g/L d-glucose (S11022, Yuanye, Shanghai, China) for 12 weeks to construct NASH mice model, and GPS (S25448, Yuanye, Shanghai, China) was given to the treatment group by oral gavage at a dose of 40 mg/kg/d as intervention therapy, while normal saline was administered daily to the HFHC and control groups. All mice could access food and water or high-sugar solution *ad libitum*. Body weights were monitored and recorded weekly. Finally, the mice were sacrificed after anesthesia to collect the liver and blood after overnight fasting. Liver tissues were either immediately snap-frozen in liquid nitrogen and then stored at −80°C or fixed in 4% paraformaldehyde (PFA, BL539A, Biosharp, China). Serum samples were obtained by centrifuging blood at 3,000 r/min at 4°C (5424R; Eppendorf, Germany).

### 2.2 Tolerance test

The glucose tolerance test (GTT) and insulin tolerance test (ITT) were measured before sacrifice. The injection volume of glucose and insulin is based on the weight of each mouse. Glucose levels were measured at time points of 0, 15, 30, 60, 90, and 120 min after intraperitoneal injection of d-glucose (1.5 g/kg) (S11022, Yuanye, Shanghai, China) and insulin injection (0.5 U/kg) (Wanbang Biopharmaceuticals, China) respectively (Sun et al., 2021).

### 2.3 Serum biochemical analysis

Serum concentrations of total triglyceride (TG), total cholesterol (TC), high-density lipoprotein cholesterol (HDL-C), low-density lipoprotein cholesterol (LDL-C), alanine aminotransferase (ALT), and aspartate aminotransaminase (AST) were determined using kits (Nanjing Jiancheng Bioengineering Institute, Nanjing, China) according to the manufacturer’s instructions. Enzyme-linked immunosorbent assay (ELISA) kits for serum tumor necrosis factor alpha (TNF-α) and transforming growth factor beta (TGF-β) were purchased from Jiangsu Meimian Industrial Co., Ltd. (Yancheng, Jiangsu, China).

### 2.4 Histological staining

Liver tissues fixed in 4% paraformaldehyde (PFA) were processed and embedded into paraffin blocks, then sliced into sections with a thickness of 0.4 μm for hematoxylin and eosin (H&E) staining, the liver specimens were sliced with neutral resin and observed under an optical microscope. The frozen sections of liver tissues (10 μm) were stained with Oil Red O according to the manufacturer’s instructions. The images were acquired and scanned by a Digital pathology scanning system (Pannoramic MIDI, 3D HISTE). The NAFLD activity score (NAS) was computed by steatosis, intralobular inflammation, and hepatocyte ballooning to assess disease severity (Brunt et al., 2011). It was calculated based on the H&E staining results using the NAS scoring system (Kleiner et al., 2005; Zeng et al., 2008). The areas of Oil Red O staining were analyzed using the software ImageJ (1.53e/java 1.8.0_172).

### 2.5 Untargeted metabolomics analysis

For extensive screening of the biomarkers that revealed the potential mechanism of GPS on NASH, we conducted the ultra-high performance liquid chromatography-Q Exactive Orbitrap-mass spectrometry (UHPLC-QE-MS). The extracted liver samples for each group (6 per group) were analyzed to obtain global metabolite profiles using an ultra-high-performance liquid chromatograph system (Vanquish, Thermo Fisher Scientific) along with Q Extractive HFX mass spectrometer (Orbitrap MS, Thermo). 25 mg of liver samples was added with 500 µL methanol/water (3:1, v/v) containing isotopically labeled internal standard mixture. Then the samples were homogenized (35 Hz, 4 min) and sonicated (5 min) in an ice-water bath three times. Afterwards, the samples were centrifuged at 12,000 rpm for 15 min at 4°C after incubating for 1 h at −20°C. The resulting supernatants (25 µL) were transferred to a fresh glass vial for analysis. The quality control (QC) sample was prepared by mixing an equal aliquot of the supernatants (10 µL) from all of the samples. The raw data were converted to mzXML format with ProteoWizard and then processed with an in-house program (developed using R for automatic data analysis) for peak detection, extraction, alignment, and integration (Biotree, Shanghai). Then an in-house MS2 database (BiotreeDB, V2.1) and HMDB database (https://hmdb.ca/) were applied to annotate the detected substances and metabolites. The metabolites with MS2 scores over 0.85 were considered as reliable results and selected for further bioinformatic analysis. Among the identified metabolites, metabolites that were significantly altered between groups (variable importance for the projection (VIP) > 1 and *p* < 0.05 in multivariate and univariate statistical analysis simultaneously) were defined as differently expressed metabolites (DEM). Principal component analysis (PCA) and orthogonal projections to latent structures-discriminant analysis (OPLS-DA) models were constructed to highlight the overall distribution trend of metabolomics profiles and the extent of sample variation between groups. A Z-score plot was performed for intuitive visualization of the distribution of each different metabolite among the different groups. Kyoto Encyclopedia of Genes and Genomes (KEGG) was performed to examine the pathway enrichment of metabolites.

### 2.6 Targeted metabolomics analysis

For validation of the biomarker identified by untargeted metabolomics, we conducted a targeted metabolomics analysis (Xie et al., 2021). Approximately 30 mg of each mouse liver tissue sample was weighed. The tissue was placed in the safe-lock tube, homogenized with 25 μL of Millipore ultrapure water, and extracted with 150 μL of cold methanol with the internal standard mix. After centrifugation (14,000 Revolutions Per Minute) at 4°C for 20 min, a 30 μL aliquot of the supernatant was carefully transferred to a 96-well plate for subsequent derivatization. The derivatization regents, 3-nitrophenylhydrazine (3-NPH) and N-(3-(dimethylamino)propyl)-N′-ethylcarbodiimide (EDC)·HCl, as well as HPLC grade solvents including methanol, ethanol, acetonitrile, formic acid, and pyridine were purchased from Sigma-Aldrich (St. Louis, MO). 20 μL of freshly prepared derivative reagents (200 mM 3-NPH in 75% aqueous methanol and 96 mM EDC-6% pyridine solution in methanol) was added to each well. The plate was sealed and the derivatization was carried out at 30°C for 60 min. After derivatization, the plate was lyophilized (Labconco, Kansas City, MO, USA) to dry. Then 400 μL of ice-cold 50% methanol solution was added to resolve the sample, followed by 4000 g centrifugation at 4°C for 30 min 140 μL of supernatant was transferred to a new 96-well plate with 10 μL internal standard mix Ⅱ in each well. Finally, the plate was sealed for liquid chromatography-mass spectrometry (LC-MS) analysis. Thereafter, we carried out statistical comparisons using the one-way analysis of variance (ANOVA) to evaluate the absolute abundance of metabolites among different groups.

### 2.7 Quantitative real-time polymerase chain reaction (qRT-PCR)

Total RNA was extracted from liver tissue samples according to protocols by TRIzol reagent (15596026, Invitrogen, United States), and the concentrations were measured using NanoPhotometer (Implen, United States). Subsequently, SYBR®Green Premix Pro Taq HS qPCR Kit (AG11701, Accurate Biology, China) was applied to conduct qRT-PCR based on QuantStudio 5 Real-Time PCR System (Thermo Fisher Scientific, United States). The relative mRNA levels of target genes were calculated by 2^−ΔΔCT^, and beta-actin was used to normalize the samples. All primer sequences used in this research are listed in Supplementary Appendix Table S1.

### 2.8 Western blotting

Total proteins were extracted from HepG2 cells or snap-frozen liver samples using the appropriate RIPA lysis buffer (P0013B, Beyotime, China). Tissue samples were ground with magnetic beads, and centrifuged at 14000 revolutions per minute for 15 min at 4°C after homogenization. The supernatant was then taken to measure the protein concentration by BCA Protein Assay Kit (P0012, Beyotime, China), and was added with loading buffer (P0015L, Beyotime, China) to heat at 100°C for 5 min after the protein concentration of each sample was adjusted. Appropriate amounts of proteins were separated by SDS-PAGE gel electrophoresis at a voltage of 80 or 120 V (Bio-Rad Laboratories, Inc., USA). Then the proteins were transferred onto polyvinylidene fluoride (PVDF) membranes (ISEQ00010, Millipore, United States) under the condition of fixed current at 270 mA for 120 min. The PVDF membranes were blocked with 5% skimmed milk (232100, BD Biosciences, United States) for over 90min, and subsequently hybridized overnight with primary antibodies at 4°C on an orbital shaker. The blots were then reacted with the secondary antibody, followed by ECL Western blotting Substrate (32,209, Invitrogen, United States). Finally, the PVDF membranes were imaged by Bio-Rad ChemiDoc XRS System (Hercules, United States). The quantitative analyses were calculated by ImageJ (1.53e/java 1.8.0_172) (National Institute of Health, United States). Detailed information about the primary and secondary antibodies was provided in Supplementary Appendix Table S2.

### 2.9 Cell culture

HepG2 cells (HB-8065, ATCC, United States) that provided by Genetimes ExCell International Holdings Limited were cultured in Dulbecco’s modified Eagle’s medium (DMEM, Gibco, United States) containing 10% fetal bovine serum (FBS, Gibco, United States) and 1% Bovine Serum Albumin (BSA, S25762, Yuanye, Shanghai, China) before intervention. HepG2 cells were seeded into 6-well plates at a density of about 2.5 × 10^5^ cells per well and incubated until the culture reached approximately 60% confluence. For the establishment of a lipotoxic cell model, palmitic acid (PA, B21705, Yuanye, Shanghai, China) that dissolved in dimethyl sulfoxide (D8418, Sigma, United States) was applied for 24 h at the concentration of 200 μM. Different concentration of glycine (Gly, B21915, Yuanye, Shanghai, China), L-serine (Ser, B21932, Yuanye, Shanghai, China), eicosapentaenoic acid (EPA, B26385, Yuanye, Shanghai, China) and docosahexaenoic acid (DHA, B27406, Yuanye, Shanghai, China) were added in the same time with PA. Finally, the cells were collected for staining and protein was extracted for Western blotting.

### 2.10 Intracellular Oil Red O staining

HepG2 cells in 6-well plates were washed with phosphate-buffered saline (PBS) before adding 4% PFA for 30 min. 60% isopropanol was added after the cells were fixed in the plates for 2 min. The oil red staining solution was mixed with diluent according to its manual (D027-1-1, Nanjing Jiancheng Bioengineering Institute, Nanjing, China). After mixing, the mixture of oil red was filtered using a microporous membrane. The mixture was then added to plates for 20 min and washed with PBS for three times. Finally, hematoxylin (ab245880, Abcam, Hongkong) was added for 5 s and images were obtained on an Olympus IX73 microscope (Tokyo, Japan). The areas of Oil Red O staining were analyzed using the software ImageJ (1.53e/java 1.8.0_172).

### 2.11 Intracellular reactive oxygen species assay

HepG2 cells in 6-well plates were washed with PBS, then stained with cell-permeant reagent 2′,7′-dichlorofluorescin diacetate (DCFH-DA) in an incubator for 45 min. DCFH-DA (S0033M, Beyotime, China) is a fluorogenic dye that detects intracellular ROS activity. After staining, the plates were washed with buffer again to clear away the residual fluorogenic dye, and ROS generation was observed under a fluorescence microscope (Tokyo, Japan) at ×20 magnification immediately. Low light conditions should be maintained to reduce photo-bleaching. The fluorescence intensity of ROS was analyzed using the software ImageJ (1.53e/java 1.8.0_172).

### 2.12 Statistical analysis

All results are given as means ± standard deviation (SD). PRISM software (V.7.00, GraphPad Software, Inc.) was used to assess statistical significance among groups via one-way analysis of variance (ANOVA) followed by post-Dunnett’s multiple comparisons tests. *p* < 0.05 was considered as significant.

## 3 Results

### 3.1 GPS treatment reduces the inflammatory response and corrects metabolic abnormalities in mice with NASH

**FIGURE 1:**
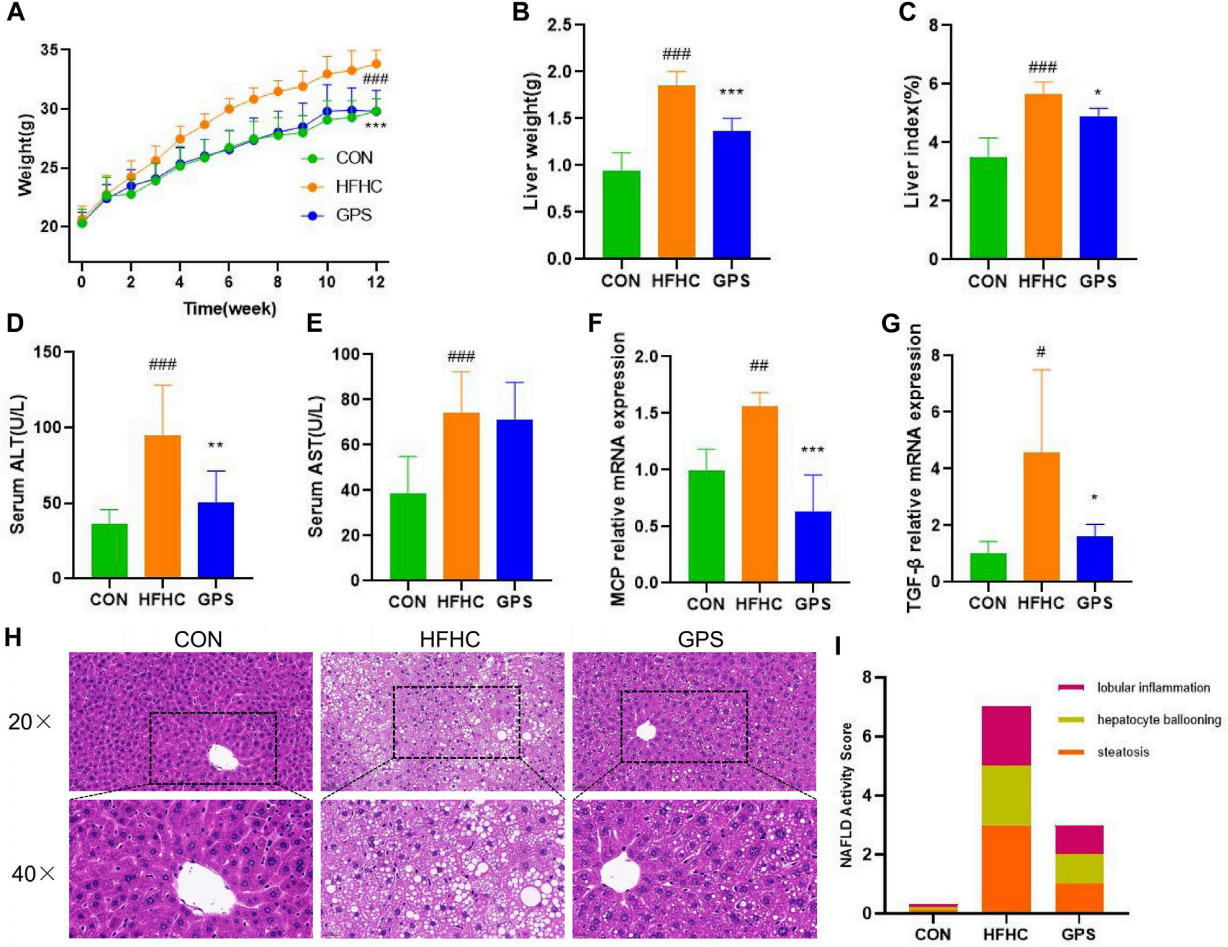
GPS supplementation attenuated the liver inflammation (n = 8 per group). **(A)** Body weight trend. **(B)** Liver weight. **(C)** liver-to-body weight ratio. **(D)** Serum ALT. **(E)** Serum AST. **(F,G)** The mRNA expression levels of MCP and TGF-β in liver tissue, and β-actin were used to normalize the samples (n = 5 per group). **(H)** histological staining of H&E. **(I)** NAFLD Activity Score. Results were presented as means ± SD. #*p* < 0.05, ##*p <* 0.01 and ###*p* < 0.001 compared with the control group, and *p < 0.05, **p < 0.01 and ***p < 0.001 compared with the HFHC group.

**FIGURE 2:**
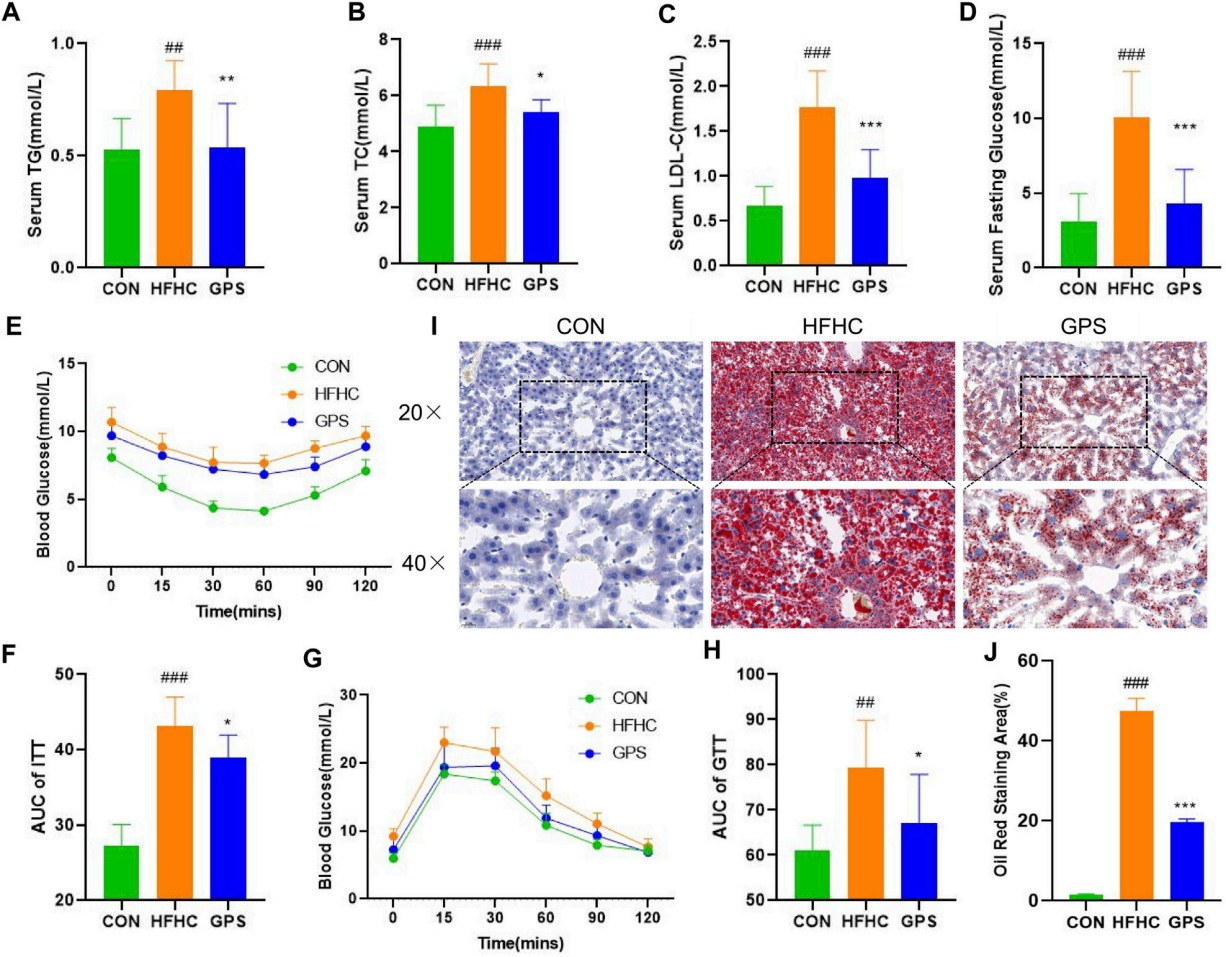
GPS supplementation attenuated the metabolic phenotypes (n = 8 per group). **(A)** Serum TG. **(B)** Serum TC. **(C)** Serum LDL-C. **(D)** Serum Fasting Glucose. **(E)** ITT. **(F)** The area under the curve of ITT. **(G)** GTT. **(H)** The area under the curve of GTT. **(I)** histological staining of Oil Red O. **(J)** Oil Red O staining area. Results were presented as means ± SD. #*p* < 0.05, ##*p <* 0.01 and ###*p* < 0.001 compared with the control group, and *p < 0.05, **p < 0.01 and ***p < 0.001 compared with the HFHC group.

In this study, mice with NASH phenotypes were induced by feeding a high-fat, high-cholesterol (HFHC) diet and high-sugar solution. Treatment with GPS significantly reduced the increase in body weight observed in the HFHC group compared to the control group, indicating that GPS may alleviate the overweight caused by the HFHC diet and high-sugar solution (Figure 1A). GPS also decreased liver weight and liver index compared to the HFHC group, suggesting that it may reduce hepatic inflammation and lipid accumulation (Figures 1B, C). Markers of inflammatory response, including serum ALT, AST, and hepatic gene expression of monocyte chemotactic protein 1 (MCP) and TGF-β, were all significantly elevated in the HFHC group but significantly decreased in the GPS group, except AST (Figures 1D–G). ELISA analysis also revealed that serum TGF-β and TNF-α were increased in the HFHC group, but TGF-β was significantly decreased in the GPS group, while TNF-α was only slightly decreased without statistical significance (Supplementary Appendix Figure S1). Metabolic indicators such as serum TG, TC, LDL-C, and fasting glucose levels were all significantly increased in the HFHC group, but significantly reduced with GPS treatment (Figures 2A–D). GPS also improved fasting blood glucose levels in the HFHC group and significantly decreased the area under the curve (AUC) for the glucose tolerance test (GTT) and insulin tolerance test (ITT) compared to the HFHC group (Figures 2E–H). GTT and ITT results showed that GPS can improve glucose tolerance and insulin resistance induced by the HFHC diet. In addition, H&E results displayed more severe pathological damages in terms of infiltration of numerous neutrophils and accumulation of lipid droplets in NASH mice induced by HFHC diet and high-sugar solution (Figure 1H). GPS distinctly reduced the NAFLD Activity Score, presenting biological functions for reducing inflammatory response (Figure 1I). Oil red O staining demonstrated that GPS significantly reduced the severe accumulation of lipid droplets caused by the HFHC diet (Figures 2I, J). The above results altogether suggested that GPS treatment provide protective effects against inflammatory response and metabolic abnormalities and improve pathological damage in mice with NASH.

### 3.2 Untargeted metabolomic analysis of liver tissues

**FIGURE 3:**
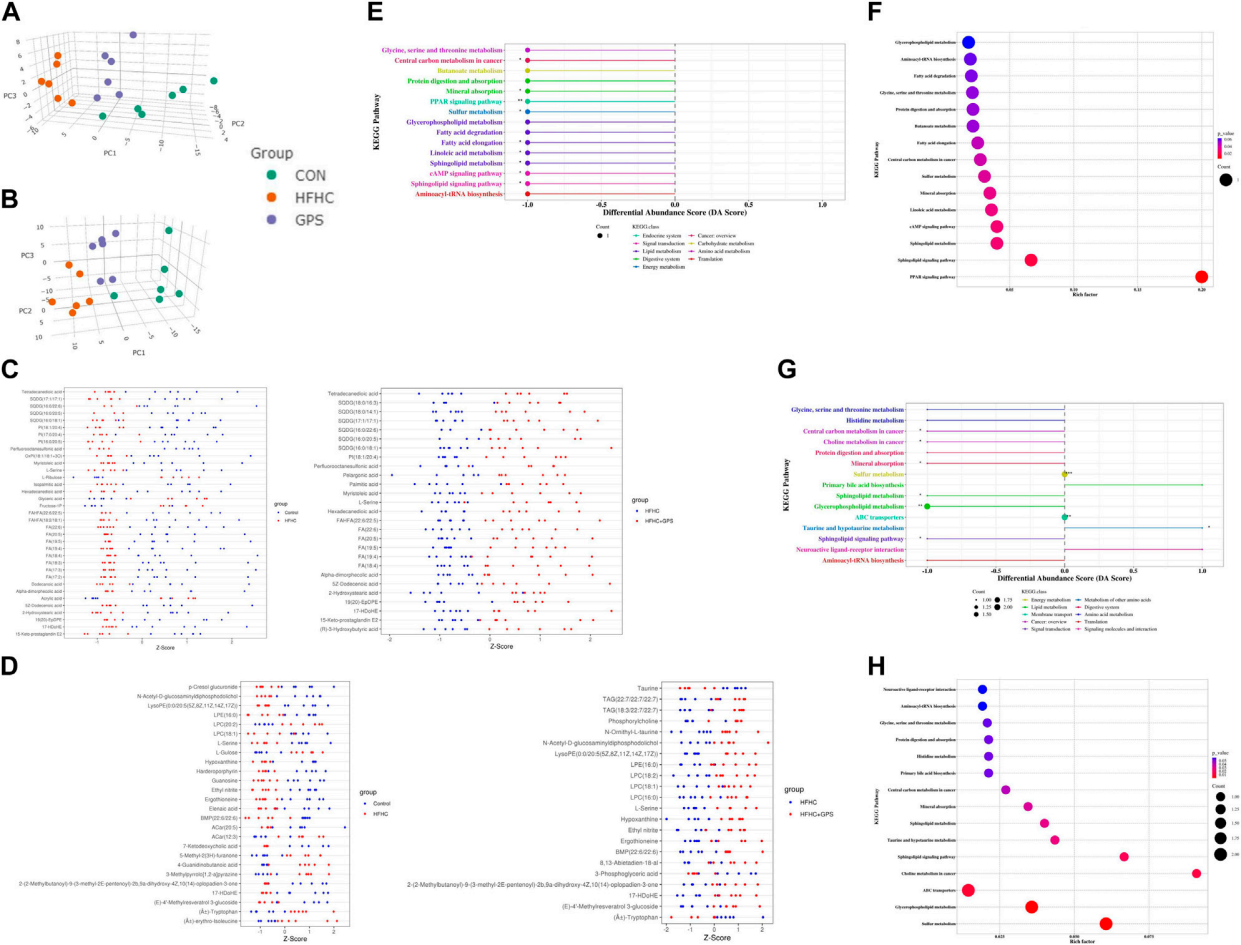
Untargeted metabolomic analysis (n = 6 per group). **(A)** 3D-PCA scores plot in Negative Ion Mode. **(B)** 3D-PCA scores plot in Positive Ion Mode. **(C)** Z-score plot in Negative Ion Mode. **(D)** Z-score plot in Positive Ion Mode. **(E)** differential abundance score between the HFHC group and GPS group in Negative Ion Mode. **(F)** bubble plot between the HFHC group and GPS group in Negative Ion Mode. **(G)** differential abundance score between the HFHC group and GPS group in Positive Ion Mode. **(H)** bubble plot between the HFHC group and GPS group in Positive Ion Mode.

In this study, untargeted metabolomic analysis of liver tissue was conducted using negative ionization mode (NIM) and positive ionization mode (PIM). The results of untargeted metabolomic analysis of liver tissues are shown in Figure 3. A total of 183 and 383 metabolites were identified according to the selection criteria (MS2 score >0.85) in NIM and PIM, respectively. 3D-principal component analysis (PCA) scores plot in NIM and PIM was generated (Figures 3A, B. PCA of these metabolites revealed that they had 54.52% and 42.54% explanatory power in NIM and PIM, respectively, indicating a clear separation among the groups in both modes and GPS treatment significantly altered the metabolic profiles of the liver tissue of mice fed a HFHC diet. To further identify the metabolites potentially linked to the therapeutic mechanism of GPS, common endogenous DEM in the liver tissue among the three groups were selected based on the cutoff criteria (VIP >1 and *p* < 0.05). Relative amounts of DEM between the two groups could be observed with a Z-score plot. In the Z-score plot, each dot represented one single sample, red and blue dots represented samples from two different groups, and the location and distance of these dots represented the relative amounts of components. A total of 35 DEMs were altered between the control group and the HFHC group, and 27 DEMs were altered between the HFHC group and the GPS group in NIM (Figure 3C). Of these, 22 DEM, including fatty acids such as eicosapentaenoic acid (EPA) and docosahexaenoic acid (DHA) and amino acids such as L-serine, were downregulated in the HFHC group and reversed by GPS. In PIM, a total of 26 DEMs were altered between the control group and the HFHC group, and 22 DEM were altered between the HFHC group and the GPS group (Figure 3D). Of these, 12 DEM were reversed by GPS, with L-serine being the common metabolite detected in both NIM and PIM. The Z-score plot result indicated that GPS reversed the alteration of DEM caused by NASH.

The KEGG pathway enrichment analysis was conducted to identify the underlying mechanisms of GPS in the treatment of NASH. In the negative ionization mode (NIM), nine KEGG pathways were found to be significantly associated with GPS supplementation, including central carbon metabolism in cancer, mineral absorption, PPAR signaling pathway, sulfur metabolism, fatty acid elongation, linoleic acid metabolism, sphingolipid metabolism, cAMP signaling pathway, and sphingolipid signaling pathway. Among these pathways, the PPAR signaling pathway had the highest enrichment factor (Figures 3E, F). Six of the nine significant KEGG pathways in NIM were also present in the differential pathways between the control group and the HFHC group (Supplementary Appendix Figure S2). These pathways were all suppressed in NASH and reversed by GPS treatment. In the positive ionization mode (PIM), seven KEGG pathways with significant differential abundance scores were enriched (Figures 3G, H). Glycine, serine, and threonine metabolism was a common pathway among the three groups and was relatively downregulated in NASH (Supplementary Appendix Figure S3).

### 3.3 Targeted metabolomic analysis on liver tissues

**FIGURE 4:**
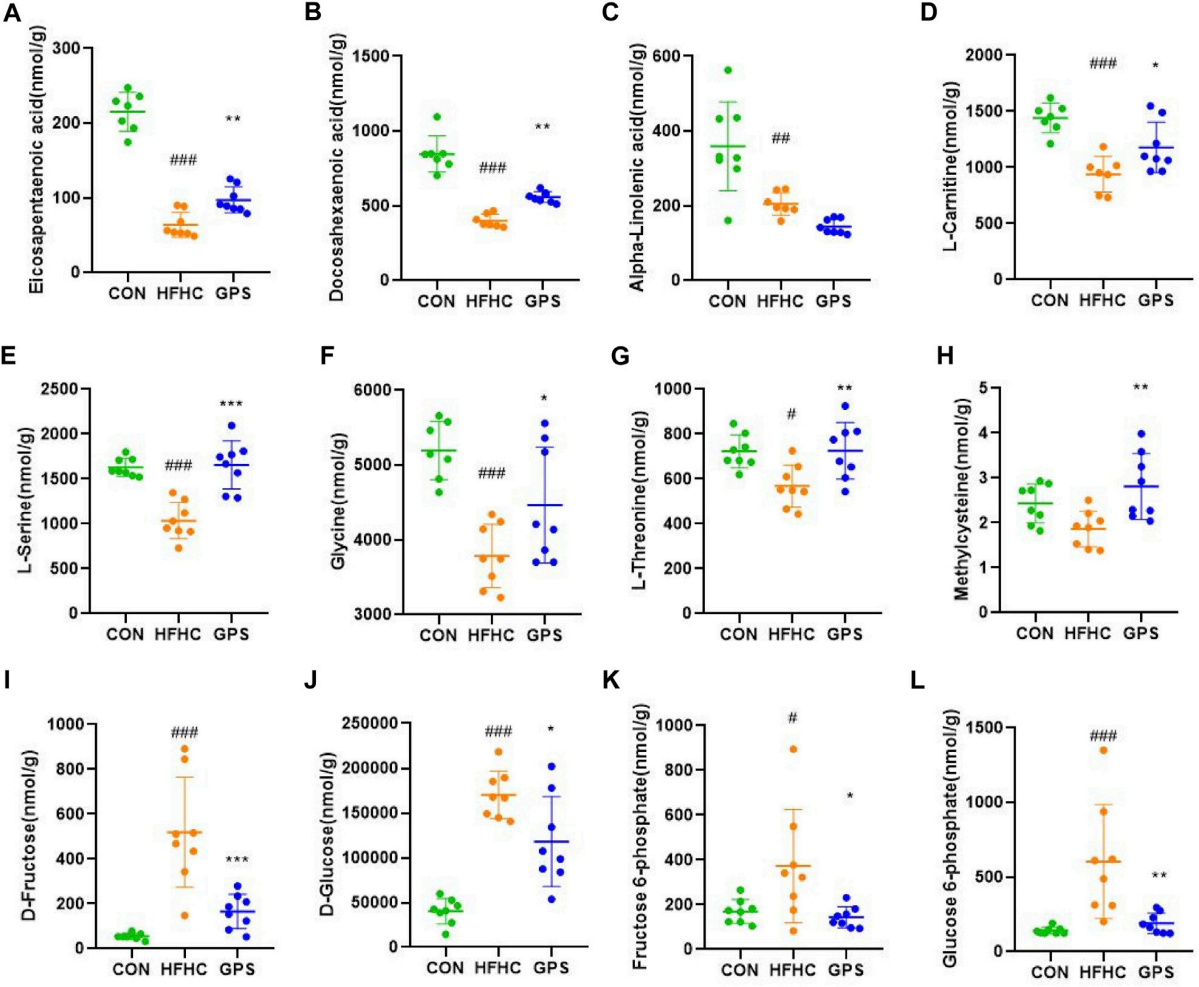
Targeted metabolomic analysis (n = 7-8 per group). **(A)** eicosapentaenoic acid. **(B)** docosahexaenoic Acid. **(C)** α-Linolenic acid. **(D)** L-carnitine. **(E)** L-serine. **(F)** glycine. **(G)** L-threonine. **(H)** Methylcysteine. **(I)** D-Fructose. **(J)** D-Glucose. **(K)** Fructose 6-phosphate. **(L)** Glucose 6-phosphate. Results were presented as means ± SD. #*p* < 0.05, ##*p <* 0.01 and ###*p* < 0.001 compared with the control group, and *p < 0.05, **p < 0.01 and ***p < 0.001 compared with the HFHC group.

The targeted metabolomic analysis method developed at Hongkong Baptist University can simultaneously identify over 200 metabolites in various samples. Our major focus is on the absolute content of fatty acids and amino acids in the liver of three groups. The results of the untargeted metabolomic analysis showed that the hepatic content of EPA and DHA was significantly decreased in NASH mice, but was increased after administration of GPS (Figures 4A, B). However, the hepatic level of another omega-3 polyunsaturated fatty acid, alpha-linolenic acid (ALA), which is a precursor of EPA and DHA, remained low in the GPS group (Figure 4C). According to previous KEGG pathway analysis and current studies, GPS may upregulate the hepatic expression of PPARα by increasing the hepatic content of EPA and DHA (Zuniga et al., 2011), as PPARα can be activated by molecules such as long-chain unsaturated fatty acids or eicosanoids. PPARα and its downstream targets are involved in the transcriptional activation of the peroxisome fatty acid beta-oxidation system, which requires the participation of carnitine (Pyper et al., 2010). Our targeted metabolomic profiles also confirmed that GPS could reverse the low level of L-carnitine in the liver of NASH mice (Figure 4D).

In terms of amino acids (AA), L-serine was the only common differentially expressed metabolite detected in the NIM and PIM. Additionally, glycine, serine, and threonine metabolism were enriched by the pathway analysis. Therefore, we focused on the variation of L-serine, glycine, and L-threonine. These amino acids are closely related to the synthesis of glutathione, a natural antioxidant that helps oppose reactive oxygen species production, scavenge existing free radicals, and repair ROS-induced damage to cell structures (Adeoye et al., 2018). The amino acids involved in glutathione synthesis are glutamate, cysteine, and glycine, but methionine and serine, which are precursors of cysteine, are also involved. Multiple studies have shown that hepatic and circulating levels of L-serine and glycine are relatively decreased during liver inflammation (Zhou et al., 2016; Mardinoglu et al., 2017; Sim et al., 2019). In line with the untargeted metabolomic analysis results, the hepatic content of L-serine, glycine, and L-threonine was significantly lower in the HFHC group and increased in the GPS group (Figures 4E–G). The level of methylcysteine in the liver of NASH mice was not decreased but significantly elevated in the GPS group (Figure 4H). Other amino acids involved in glutathione synthesis, such as L-glutamic acid and L-methionine, were also reduced in the HFHC group, but only L-methionine was reversed in the GPS group (Supplementary Appendix Figure S4).

Additionally, the targeted metabolomic profiling results showed that GPS intervention significantly reduced the elevated levels of D-fructose, D-glucose, fructose 6-phosphate, and glucose 6-phosphate in the HFHC diet (Figures 4I, J).

### 3.4 GPS improved the condition of NASH mice through activation of the PPARα pathway and suppression of the HIF-1α pathway

**FIGURE 5:**
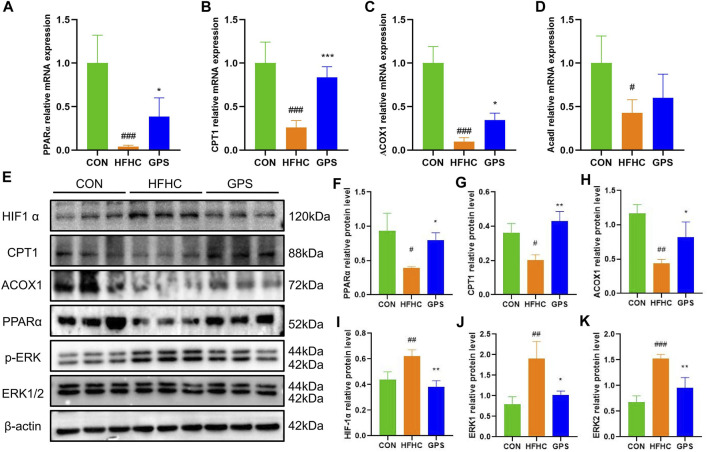
GPS enhanced the PPARα signaling pathway and inhibited the HIF-1α signaling pathway in mice. **(A)** The mRNA expression levels of PPARα in liver tissue and β-actin were used to normalize the samples (n = 4-5 per group). **(B)** The mRNA expression levels of CPT1 in liver tissue and β-actin were used to normalize the samples (n = 4-5 per group). **(C)** The mRNA expression levels of ACOX1 in liver tissue and β-actin were used to normalize the samples (n = 4-5 per group). **(D)** The mRNA expression levels of Acadl in liver tissue and β-actin were used to normalize the samples (n = 4-5 per group). **(E)** Western blot analysis of PPARα, CPT1, ACOX1 HIF-1α, p-ERK/ERK1, and p-ERK/ERK2 in liver tissues (n = 3 per group). **(F)** Statistical graph of PPARα protein level in liver tissues. **(G)** Statistical graph of CPT1 protein level in liver tissues. **(H)** Statistical graph of ACOX1 protein level in liver tissues. **(I)** Statistical graph of HIF-1α protein level in liver tissues. **(J)** Statistical graph of p-ERK/ERK1 protein level in liver tissues. **(K)** Statistical graph of p-ERK/ERK2 protein level in liver tissues. Results were presented as means ± SD. #*p* < 0.05, ##*p <* 0.01 and ###*p* < 0.001 compared with the control group, and *p < 0.05, **p < 0.01 and ***p < 0.001 compared with the HFHC group.

The PPARα is a transcription factor that regulates the expression of genes encoding enzymes involved in fatty acid oxidation (FAO). These include carnitine palmitoyltransferase 1 (CPT1) and acyl-coenzyme A oxidase 1 (ACOX1), which allow fatty acids to enter the β-oxidation pathway. In this study, the transcriptional expression of PPARα, CPT1, and ACOX1 was significantly decreased in the liver tissues of mice fed with HFHC diet and high-sugar solution. However, treatment with GPS significantly reversed this trend (Figures 5A–C). The expression of Acyl-Coenzyme A Dehydrogenase, Long Chain (ACADL) was also reduced in the HFHC group, but the effect of GPS on ACADL was slight (Figure 5D). Western blot analysis also showed that the protein levels of PPARα, CPT1, and ACOX1 were significantly suppressed in the HFHC group, but were significantly reversed by GPS treatment, indicating that GPS can activate the PPARα signaling pathway to reduce inflammation and facilitate the β-oxidation of non-esterified fatty acids (NEFAs) (Figures 5E–H). The accumulation of mitochondrial reactive oxygen species (ROS) activates oxidative stress (OS), which results in mitochondrial dysfunction and liver inflammation (Rives et al., 2020; Zhao et al., 2021). ROS seems to enhance the signaling activity of extracellular signal-regulated kinases (ERK), which in turn induce Hypoxia-Inducible Factor-1α (HIF-1α) transcription and translation (Movafagh et al., 2015). Here we also found that the protein levels of HIF1-α and the phosphorylation levels of ERK1/2 were significantly increased in the HFHC group, but significantly decreased by GPS treatment (Figures 5I–K).

### 3.5 EPA and DHA regulated PPARα to promote fatty acid oxidation

**FIGURE 6:**
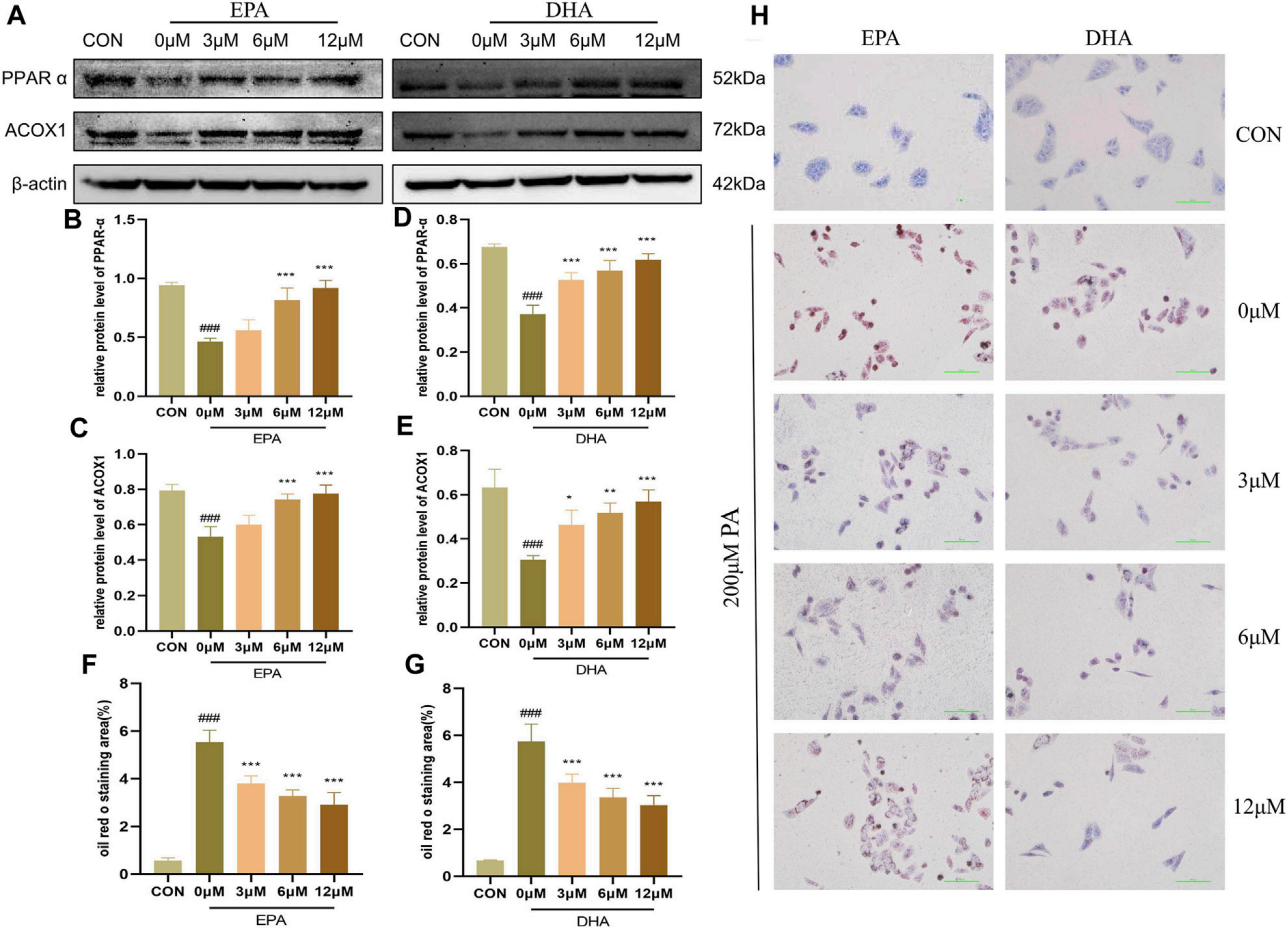
EPA and DHA regulated PPARα to promote fatty acid oxidation (n = 3 per group). **(A)** Western blot analysis of PPARα and ACOX1 with different concentrations of EPA and DHA supplementation. **(B)** Statistical graph of PPARα protein level after EPA supplementation. **(C)** Statistical graph of ACOX1 protein level after EPA supplementation. **(D)** Statistical graph of PPARα protein level after DHA supplementation. **(E)** Statistical graph of ACOX1 protein level after DHA supplementation. **(F)** Intracellular oil Red O staining area of EPA supplementation. **(G)** Intracellular oil Red O staining area of DHA supplementation. **(H)** Intracellular Oil Red O staining. Results are presented as means ± SD. #*p* < 0.05, ##*p <* 0.01 and ###*p* < 0.001 compared with the control group, and *p < 0.05, **p < 0.01 and ***p < 0.001 compared with the HFHC group.

To investigate the potential role of EPA and DHA in the molecular mechanisms of GPS on NASH, we performed *in vitro* experiments to examine the effects of these fatty acids on fatty acid oxidation (FAO) in HepG2 cells. We hypothesized that EPA and DHA would regulate the PPARα signaling pathway to enhance FAO. To further investigate the impact of EPA and DHA on the PPARα signaling pathway, we performed Western blot analyses and found that the protein levels of PPARα and ACOX were suppressed by 200 μM PA treatment, but were restored after treatment with different concentrations of EPA and DHA (Figures 6A–E). Oil red O staining showed that 200 μM PA treatment led to an increase in the accumulation of lipid droplets in HepG2 cells, while different concentrations of EPA and DHA (3.125μM–12.5 μM) effectively reduced this trend, indicating an anti-steatosis effect of EPA and DHA (Figures 6F–H). These results support the hypothesis that EPA and DHA can enhance FAO. These results suggest that EPA and DHA may regulate PPARα signaling to enhance FAO in NASH.

### 3.6 L-serine and glycine regulated HIF-1α to inhibit oxidative stress

**FIGURE 7:**
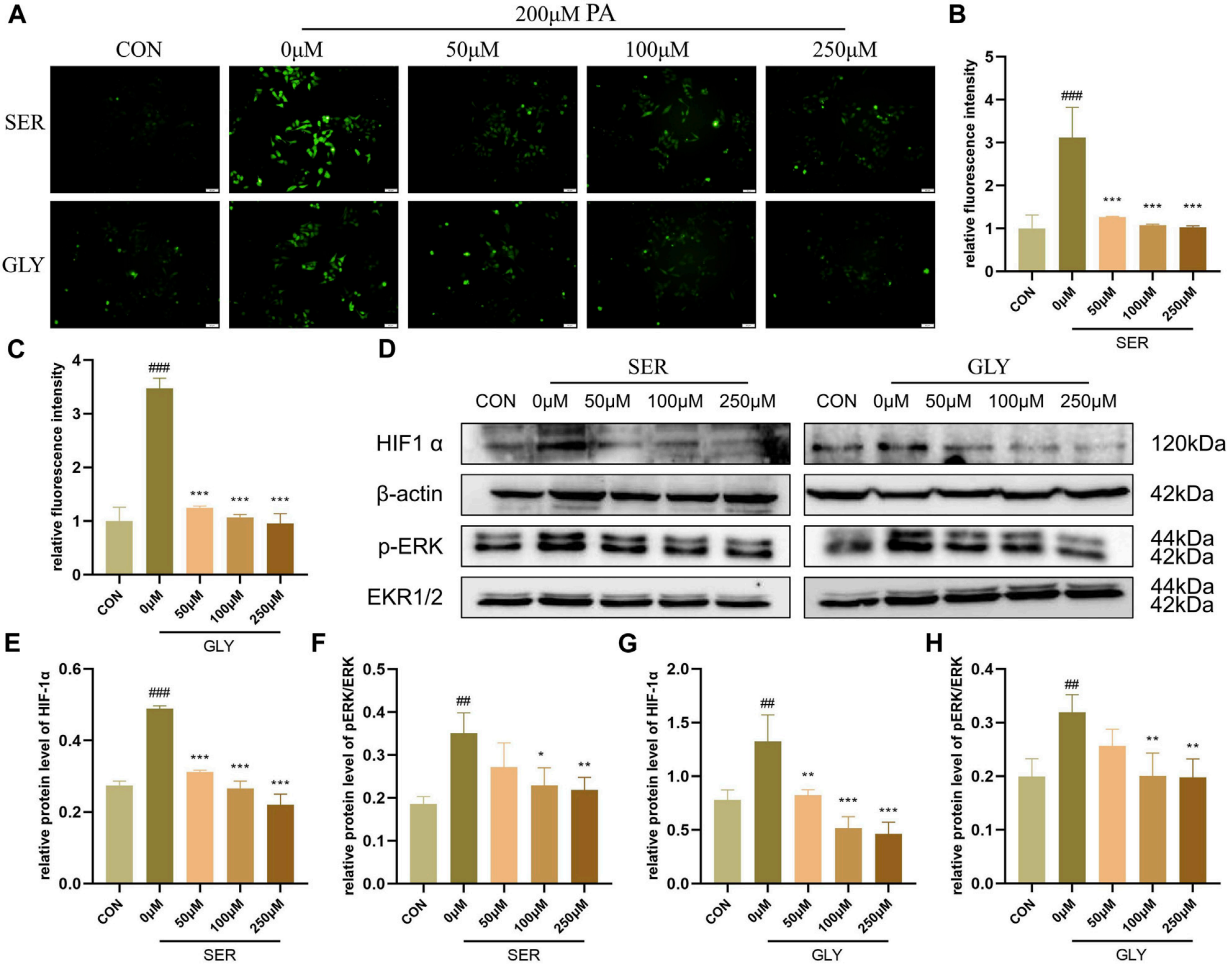
L-serine and glycine regulated HIF1α to inhibit oxidative stress (n = 3 per group). **(A)** Intracellular ROS level with different concentrations of L-serine and glycine supplementation. **(B)** Statistical graph of fluorescence intensity after L-serine supplementation. **(C)** Statistical graph of fluorescence intensity after glycine supplementation. **(D)** Western blot analysis of HIF1α and phosphorylation level of ERK with different concentrations of L-serine and glycine supplementation. **(E)** Statistical graph of HIF1α protein level after L-serine supplementation. **(F)** Statistical graph of phosphorylation level of ERK after L-serine supplementation. **(G)** Statistical graph of HIF1α protein level after glycine supplementation. **(H)** Statistical graph of the phosphorylation level of ERK after glycine supplementation. Results are presented as means ± SD. #*p* < 0.05, ##*p <* 0.01 and ###*p* < 0.001 compared with the control group, and *p < 0.05, **p < 0.01 and ***p < 0.001 compared with the HFHC group.

A significant amount of literature suggests that hypoxia-inducible factor-1 alpha (HIF-1α) plays a direct regulatory role in ROS, although the existing literature contains contradictory findings (Movafagh et al., 2015). However, overexpression of the HIF subunit induced by hypoxic conditions worsens NAFLD pathology by suppressing fatty acid oxidation (FAO)-related genes such as PPARα, CPT1α, and ACOX1, leading to mitochondrial impairment and dysfunction in the liver (Chen et al., 2019). The deficiency of HIF-1α in hepatocytes abolishes the reduction of the protein levels of FAO-related genes (Liu et al., 2014). Therefore, we hypothesized that L-serine and glycine improve the inflammatory response of NASH through the HIF-1α pathway. Our fluorescence staining results revealed that green fluorescence intensity, which represents the intracellular level of ROS, was significantly increased in PA-stimulated hepatocytes and was dramatically alleviated with the addition of L-serine and glycine (Figures 7A–C). Western blot analysis showed that the protein level of HIF-1α and the phosphorylation levels of ERK were all increased in the PA group, but significantly suppressed by L-serine and glycine, indicating that L-serine and glycine can inhibit this signaling to restrain OS and further impede the inflammatory cascade (Figures 7D–H).

## 4 Discussion

This study showed that GPS is effective in improving the phenotype of HFHC-induced NASH mice, including reducing body weight gain and liver weight, decreasing serum levels of ALT, TG, TC, and LDL-C, and improving insulin sensitivity, as well as attenuating hepatic steatosis. HFHC diet and high-sugar solution were confirmed to be a feasible method to establish NASH mice (Zhang X. et al., 2021). We used untargeted and targeted metabolomics to confirm the molecular mechanisms of GPS on NASH. We found that some omega-3 Polyunsaturated fatty acids (omega-3 PUFAs), such as EPA and DHA, as well as various amino acids, such as L-serine and glycine, were significantly decreased in the liver of NASH mice and reversed by GPS. The KEGG annotation results showed that GPS supplementation activates the PPARα signaling pathway. We also showed that GPS promotes the recovery of EPA and DHA to facilitate fatty acid oxidation, and modulates the homeostasis of L-serine and glycine to protect mitochondrial function and reduce oxidative stress.

It is not unique that GPS is effective on NASH, accumulating evidence demonstrated that GPS could not only improve NAFLD but also encompass a wide range of effects in other metabolic diseases (Liu et al., 2014). GPS significantly attenuated glucose tolerance, insulin resistance, and dyslipidemia, reduced the inflammatory response in liver injury, diabetes mellitus, and diabetic retinopathy (Liu et al., 2017; Xiao et al., 2022; Zou et al., 2022; Wang et al., 2023; Zhang et al., 2023). However, the mechanisms underlying the hepatoprotective effects of GPS are not well understood, hence, we used untargeted and targeted metabolomics to investigate whether the benefits of GPS are associated with the modulation of internal metabolism. The novelty of this study was revealing the liver metabolic profile in NASH mice treated with GPS, and the anti-NASH mechanism of GPS through the alteration in hepatic metabolites. The combined metabolomics results showed that numerous PUFAs and amino acids were dramatically altered in the HFHC group and reversed by GPS.

PUFAs are important for human health. Previous studies have shown that high intake of PUFAs is closely linked to improved glucose homeostasis and reduced cardiovascular disease risk. Omega-3 PUFAs, including ALA, EPA, and DHA (Shahidi and Ambigaipalan, 2018), have received attention due to their multiple benefits, including antioxidant, anti-inflammatory, and improved effects on diabetes and cardiovascular disease. Omega-3 PUFAs are important components of membrane phospholipids, which mediate changes in cell function by serving as a precursor pool for lipid mediators (Dyall et al., 2022). Several lipidomic profiling studies have shown that EPA and DHA are decreased in NASH (Kalhan et al., 2011; Dyall et al., 2022). EPA has been shown to have beneficial effects in HFD-induced liver steatosis and decline in energy metabolism (Echeverria et al., 2019), possibly through its interaction with PPARα (Sugiyama et al., 2008). A marked decline of DHA has been observed in the liver tissue of patients with cirrhosis, and dietary supplementation of DHA has liver protective effects (Kudrin et al., 1988; Sugiyama et al., 2008). DHA and EPA have also been shown to have an “anti-obesity' effect and prevent insulin resistance (Li et al., 2008). Consistent with these previous studies, our results also showed that the hepatic content of EPA and DHA was significantly decreased in NASH mice and markedly reversed after GPS supplementation. Western blot analysis and qPCR showed that GPS upregulated PPARα and its downstream targets. PPARα is abundantly expressed in the liver, where it is one of the key transcription factors involved in ketogenesis regulation and plays a significant role in regulating lipid metabolism, glucose homeostasis, and inflammation (Lefebvre et al., 2006; Derosa et al., 2018). Ketogenesis requires efficient mitochondrial beta-oxidation of fatty acids. Fatty acids that are taken up by the liver are either oxidized to produce energy and ketone bodies or re-esterified into TG and stored as lipid droplets. Therefore, promoting beta-oxidation of fatty acids in the mitochondria can reduce the accumulation of lipid droplets. Several studies have shown that EPA and/or DHA can reduce lipid accumulation in adipose tissue and alleviate liver cell inflammation by up-regulating PPARα and its downstream genes CPT/ACOX or by promoting the formation of PPARα/NF-κB complexes (Zuniga et al., 2011; Albracht-Schulte et al., 2019; Soni et al., 2019). Our *in vitro* experiment also showed that EPA and DHA stimulate the expression of PPARα and its target proteins to decrease intracellular lipid accumulation.

Amino acids are essential for maintaining human physiological functions (Zuniga et al., 2011). They not only act as cell signaling molecules and regulators of gene expression and the protein phosphorylation cascade but also serve as building blocks for hormone synthesis. Studies have shown that specific amino acid patterns, characterized by decreased glycine and serine levels, may be used for early detection of NAFLD and noninvasive assessment of its histological severity (Trico et al., 2021). Fasting levels of glycine and serine are decreased in NAFLD, both in human plasma and liver biopsy samples (Hyotylainen et al., 2016; Mardinoglu et al., 2017). Decreased serine levels are closely associated with the development of fatty liver through clinical and animal experiments, and serine treatment improves NAFLD by promoting L-serine-dependent homocysteine metabolism (Sim et al., 2015). In agreement with these previous reports, our results also showed that the hepatic content of L-serine and glycine was lower in NASH mice and was reversed by GPS supplementation. Serine and glycine concentrations are related to the severity of NAFLD because they are involved in glutathione (GSH) synthesis in response to OS (Gaggini et al., 2018).

Several studies have suggested that an increase in OS in the liver is associated with liver damage and the progression of NAFLD to NASH. The increase in OS results in the consumption of the major intracellular antioxidant, GSH, leading to a reduction in hepatic GSH levels (Muriel, 2009). Decreased GSH synthesis is therefore highly correlated with the development of NAFLD (Sutti et al., 2014; Honda et al., 2017). OS, defined as an imbalance of pro- and antioxidants, is harmful to cells due to the excessive generation of ROS and reactive nitrogen species (RNS) (Daenen et al., 2019). Excessive generation of ROS increases the activation of HIF-1α and NF-κB signaling pathways (Korbecki et al., 2021). Suppressing HIF-1α would be beneficial for NASH, as it controls the transcription of pro-inflammatory mediators (Zheng et al., 2021b). It has been confirmed that HIF activation decreases PPARα and its target genes involved in fatty acid oxidation in the liver, meaning that inhibiting the HIF signaling pathway would enhance the PPARα signaling pathway (Mooli et al., 2021). Our *in vivo* experiment showed that GPS downregulated the expression of HIF-1α, and our *in vitro* experiment showed that L-serine and glycine also inhibited the HIF-1α signaling pathway to reduce intracellular ROS levels.

However, there are still some limitations in this study. First, we only extracted liver tissues to perform metabolomics analysis. It would be better to implement this method in multiple types of samples to draw a more comprehensive conclusion. Another limitation of this study involved the issue of drug concentration gradient. The reason that we implemented one dosage GPS (40 mg/kg/day) was based on the existing literature and the preliminary experiment we conducted (Liu et al., 2014; Choi et al., 2019). However, it would be better to apply multiple concentration gradients to provide more reliable evidence. Last but not least, considering GPS will be translated as a potential treatment option for NASH patients someday due to its water-solubility and hepatoprotectivity, it would be better to initiate GPS treatment after disease establishment in order to provide reference value for translational medicine in real-world scenarios.

## 5 Conclusion

GPS treatment simultaneously inhibit inflammation and restore metabolic abnormalities in NASH mice induced by a HFHC diet and the high-sugar solution. Using targeted and untargeted metabolomic analysis, we discovered that GPS regulated EPA and DHA to improve metabolic disorders through activation of the PPARα pathway, and L-serine and glycine to inhibit inflammation and oxidative stress through suppression of the HIF-1α pathway.

## Data Availability

The original contributions presented in the study are included in the article/Supplementary Material, further inquiries can be directed to the corresponding authors.

## References

[B1] Adeoye O. Olawumi J. Opeyemi A. Christiania O. (2018). Review on the role of glutathione on oxidative stress and infertility. JBRA Assist. Reprod. 22 (1), 61–66. 10.5935/1518-0557.20180003 29266896 PMC5844662

[B2] Albracht-Schulte K. Gonzalez S. Jackson A. Wilson S. Ramalingam L. Kalupahana N. S. (2019). Eicosapentaenoic acid improves hepatic metabolism and reduces inflammation independent of obesity in high-fat-fed mice and in HepG2 cells. Nutrients 11 (3), 599. 10.3390/nu11030599 30871035 PMC6471632

[B3] Brunt E. M. Kleiner D. E. Wilson L. A. Belt P. Neuschwander-Tetri B. A. NASH Clinical Research Network CRN (2011). Nonalcoholic fatty liver disease (NAFLD) activity score and the histopathologic diagnosis in NAFLD: distinct clinicopathologic meanings. Hepatology 53 (3), 810–820. 10.1002/hep.24127 21319198 PMC3079483

[B4] Bujak R. Struck-Lewicka W. Markuszewski M. J. Kaliszan R. (2015). Metabolomics for laboratory diagnostics. J. Pharm. Biomed. Anal. 113, 108–120. 10.1016/j.jpba.2014.12.017 25577715

[B5] Chen J. Chen J. Fu H. Li Y. Wang L. Luo S. (2019). Hypoxia exacerbates nonalcoholic fatty liver disease via the HIF-2α/PPARα pathway. Am. J. Physiol. Endocrinol. Metab. 317 (4), E710–E722. 10.1152/ajpendo.00052.2019 31430204

[B6] Cheng Z. Song H. Zhang Y. Han D. Yu X. Shen Q. (2019). Concurrent extraction and purification of gentiopicroside from Gentiana scabra bunge using microwave-assisted ethanol-salt aqueous two-phase systems. J. Chromatogr. Sci. 58 (1), 60–74. 10.1093/chromsci/bmz101 31845984

[B7] Chin R. M. Fu X. Pai M. Y. Vergnes L. Hwang H. Deng G. (2014). The metabolite α-ketoglutarate extends lifespan by inhibiting ATP synthase and TOR. Nature 510 (7505), 397–401. 10.1038/nature13264 24828042 PMC4263271

[B8] Choi R. Y. Nam S. J. Lee H. I. Lee J. Leutou A. S. Ri Ham J. (2019). Gentiopicroside isolated from Gentiana scabra Bge. inhibits adipogenesis in 3T3-L1 cells and reduces body weight in diet-induced obese mice. Bioorg Med. Chem. Lett. 29 (14), 1699–1704. 10.1016/j.bmcl.2019.05.038 31130265

[B9] Daenen K. Andries A. Mekahli D. Van Schepdael A. Jouret F. Bammens B. (2019). Oxidative stress in chronic kidney disease. Pediatr. Nephrol. 34 (6), 975–991. 10.1007/s00467-018-4005-4 30105414

[B10] Derosa G. Sahebkar A. Maffioli P. (2018). The role of various peroxisome proliferator-activated receptors and their ligands in clinical practice. J. Cell Physiol. 233 (1), 153–161. 10.1002/jcp.25804 28098353

[B11] Dyall S. C. Balas L. Bazan N. G. Brenna J. T. Chiang N. da Costa Souza F. (2022). Polyunsaturated fatty acids and fatty acid-derived lipid mediators: recent advances in the understanding of their biosynthesis, structures, and functions. Prog. Lipid Res. 86, 101165. 10.1016/j.plipres.2022.101165 35508275 PMC9346631

[B12] Echeverria F. Valenzuela R. Bustamante A. Álvarez D. Ortiz M. Espinosa A. (2019). High-fat diet induces mouse liver steatosis with a concomitant decline in energy metabolism: attenuation by eicosapentaenoic acid (EPA) or hydroxytyrosol (HT) supplementation and the additive effects upon EPA and HT co-administration. Food Funct. 10 (9), 6170–6183. 10.1039/c9fo01373c 31501836

[B13] Gaggini M. Carli F. Rosso C. Buzzigoli E. Marietti M. Della Latta V. (2018). Altered amino acid concentrations in NAFLD: impact of obesity and insulin resistance. Hepatology 67 (1), 145–158. 10.1002/hep.29465 28802074

[B14] German J. B. Hammock B. D. Watkins S. M. (2005). Metabolomics: building on a century of biochemistry to guide human health. Metabolomics 1 (1), 3–9. 10.1007/s11306-005-1102-8 16680201 PMC1457093

[B15] Honda Y. Kessoku T. Sumida Y. Kobayashi T. Kato T. Ogawa Y. (2017). Efficacy of glutathione for the treatment of nonalcoholic fatty liver disease: an open-label, single-arm, multicenter, pilot study. BMC Gastroenterol. 17 (1), 96. 10.1186/s12876-017-0652-3 28789631 PMC5549431

[B16] Hutchison A. L. Tavaglione F. Romeo S. Charlton M. (2023). Endocrine aspects of metabolic dysfunction-associated steatotic liver disease (MASLD): beyond insulin resistance. J. Hepatol. 79 (6), 1524–1541. 10.1016/j.jhep.2023.08.030 37730124

[B17] Hyotylainen T. Jerby L. Petäjä E. M. Mattila I. Jäntti S. Auvinen P. (2016). Genome-scale study reveals reduced metabolic adaptability in patients with non-alcoholic fatty liver disease. Nat. Commun. 7, 8994. 10.1038/ncomms9994 26839171 PMC4742839

[B18] Jin M. Feng H. Wang Y. Yan S. Shen B. Li Z. (2020). Gentiopicroside ameliorates oxidative stress and lipid accumulation through nuclear factor erythroid 2-related factor 2 activation. Oxid. Med. Cell Longev. 2020, 2940746. 10.1155/2020/2940746 32655764 PMC7317617

[B19] Kalhan S. C. Guo L. Edmison J. Dasarathy S. McCullough A. J. Hanson R. W. (2011). Plasma metabolomic profile in nonalcoholic fatty liver disease. Metabolism 60 (3), 404–413. 10.1016/j.metabol.2010.03.006 20423748 PMC2950914

[B20] Kapoore R. V. Vaidyanathan S. (2016). Towards quantitative mass spectrometry-based metabolomics in microbial and mammalian systems. Philos. Trans. A Math. Phys. Eng. Sci., 374, 20150363. 10.1098/rsta.2015.0363(2079) 27644979 PMC5031630

[B21] Kleiner D. E. Brunt E. M. Van Natta M. Behling C. Contos M. J. Cummings O. W. (2005). Design and validation of a histological scoring system for nonalcoholic fatty liver disease. Hepatology 41 (6), 1313–1321. 10.1002/hep.20701 15915461

[B22] Korbecki J. Simińska D. Gąssowska-Dobrowolska M. Listos J. Gutowska I. Chlubek D. (2021). Chronic and cycling hypoxia: drivers of cancer chronic inflammation through HIF-1 and NF-κB activation: a review of the molecular mechanisms. Int. J. Mol. Sci. 22 (19), 10701. 10.3390/ijms221910701 34639040 PMC8509318

[B23] Kudrin A. N. Smolenskiĭ V. S. Kogan A. K. Abinder A. A. Khusainov V. M. (1988). Antioxidants in the treatment of experimental myocardial ischemia and ischemic heart disease. Kardiologiia 28 (7), 115–121. 3062223

[B24] Le M. H. Yeo Y. H. Li X. Li J. Zou B. Wu Y. (2019). 2019 global NAFLD prevalence: a systematic review and meta-analysis. Clin. Gastroenterol. Hepatol. 20(12). 2809–2817. 10.1016/j.cgh.2021.12.002 34890795

[B25] Lefebvre P. Chinetti G. Fruchart J. C. Staels B. (2006). Sorting out the roles of PPAR alpha in energy metabolism and vascular homeostasis. J. Clin. Invest. 116 (3), 571–580. 10.1172/JCI27989 16511589 PMC1386122

[B26] Li J. J. Huang C. J. Xie D. (2008). Anti-obesity effects of conjugated linoleic acid, docosahexaenoic acid, and eicosapentaenoic acid. Mol. Nutr. Food Res. 52 (6), 631–645. 10.1002/mnfr.200700399 18306430

[B27] Li X. Zhang Y. Jin Q. Xia K. L. Jiang M. Cui B. W. (2018). Liver kinase B1/AMP-activated protein kinase-mediated regulation by gentiopicroside ameliorates P2X7 receptor-dependent alcoholic hepatosteatosis. Br. J. Pharmacol. 175 (9), 1451–1470. 10.1111/bph.14145 29338075 PMC5900996

[B28] Liu L. Zuo Z. Lu S. Liu A. Liu X. (2017). Naringin attenuates diabetic retinopathy by inhibiting inflammation, oxidative stress and NF-κB activation *in vivo* and *in vitro* . Iran. J. Basic Med. Sci. 20 (7), 813–821. 10.22038/IJBMS.2017.9017 28852447 PMC5569591

[B29] Liu Y. Ma Z. Zhao C. Wang Y. Wu G. Xiao J. (2014). HIF-1α and HIF-2α are critically involved in hypoxia-induced lipid accumulation in hepatocytes through reducing PGC-1α-mediated fatty acid β-oxidation. Toxicol. Lett. 226 (2), 117–123. 10.1016/j.toxlet.2014.01.033 24503013

[B30] Mardinoglu A. Bjornson E. Zhang C. Klevstig M. Söderlund S. Ståhlman M. (2017). Personal model-assisted identification of NAD(+) and glutathione metabolism as intervention target in NAFLD. Mol. Syst. Biol. 13 (3), 916. 10.15252/msb.20167422 28254760 PMC5371732

[B31] Mooli R. G. R. Rodriguez J. Takahashi S. Solanki S. Gonzalez F. J. Ramakrishnan S. K. (2021). Hypoxia via ERK signaling inhibits hepatic PPARα to promote fatty liver. Cell Mol. Gastroenterol. Hepatol. 12 (2), 585–597. 10.1016/j.jcmgh.2021.03.011 33798787 PMC8258975

[B32] Movafagh S. Crook S. Vo K. (2015). Regulation of hypoxia-inducible factor-1a by reactive oxygen species: new developments in an old debate. J. Cell Biochem. 116 (5), 696–703. 10.1002/jcb.25074 25546605

[B33] Muriel P. (2009). Role of free radicals in liver diseases. Hepatol. Int. 3 (4), 526–536. 10.1007/s12072-009-9158-6 19941170 PMC2790593

[B34] Ng C. H. Huang D. Q. Nguyen M. H. (2022). Nonalcoholic fatty liver disease versus metabolic-associated fatty liver disease: prevalence, outcomes, and implications of a change in name. Clin. Mol. Hepatol. 28 (4), 790–801. 10.3350/cmh.2022.0070 35545437 PMC9597238

[B35] Pyper S. R. Viswakarma N. Yu S. Reddy J. K. (2010). PPARalpha: energy combustion, hypolipidemia, inflammation and cancer. Nucl. Recept Signal 8, e002. 10.1621/nrs.08002 20414453 PMC2858266

[B36] Rinella M. E. Lazarus J. V. Ratziu V. Francque S. M. Sanyal A. J. Kanwal F. (2023). A multisociety Delphi consensus statement on new fatty liver disease nomenclature. J. Hepatol. 79 (6), 1542–1556. 10.1016/j.jhep.2023.06.003 37364790

[B37] Rives C. Fougerat A. Ellero-Simatos S. Loiseau N. Guillou H. Gamet-Payrastre L. (2020). Oxidative stress in NAFLD: role of nutrients and food contaminants. Biomolecules 10 (12), 1702. 10.3390/biom10121702 33371482 PMC7767499

[B38] Shahidi F. Ambigaipalan P. (2018). Omega-3 polyunsaturated fatty acids and their health benefits. Annu. Rev. Food Sci. Technol. 9, 345–381. 10.1146/annurev-food-111317-095850 29350557

[B39] Sharma A. Anand S. K. Singh N. Dwivedi U. N. Kakkar P. (2020). Berbamine induced AMPK activation regulates mTOR/SREBP-1c axis and Nrf2/ARE pathway to allay lipid accumulation and oxidative stress in steatotic HepG2 cells. Eur. J. Pharmacol. 882, 173244. 10.1016/j.ejphar.2020.173244 32526241

[B40] Sim W. C. Kim D. G. Lee W. Sim H. Choi Y. J. Lee B. H. (2019). Activation of SIRT1 by L-serine increases fatty acid oxidation and reverses insulin resistance in C2C12 myotubes. Cell Biol. Toxicol. 35 (5), 457–470. 10.1007/s10565-019-09463-x 30721374

[B41] Sim W. C. Yin H. Q. Choi H. S. Choi Y. J. Kwak H. C. Kim S. K. (2015). L-serine supplementation attenuates alcoholic fatty liver by enhancing homocysteine metabolism in mice and rats. J. Nutr. 145 (2), 260–267. 10.3945/jn.114.199711 25644346

[B42] Soni N. Ross A. B. Scheers N. Nookaew I. Gabrielsson B. G. Sandberg A. S. (2019). The omega-3 fatty acids EPA and DHA, as a part of a murine high-fat diet, reduced lipid accumulation in Brown and white adipose tissues. Int. J. Mol. Sci. 20 (23), 5895. 10.3390/ijms20235895 31771283 PMC6928976

[B43] Sugiyama E. Ishikawa Y. Li Y. Kagai T. Nobayashi M. Tanaka N. (2008). Eicosapentaenoic acid lowers plasma and liver cholesterol levels in the presence of peroxisome proliferators-activated receptor alpha. Life Sci. 83 (1-2), 19–28. 10.1016/j.lfs.2008.04.011 18541273

[B44] Sun N. Shen C. Zhang L. Wu X. Yu Y. Yang X. (2021). Hepatic Krüppel-like factor 16 (KLF16) targets PPARα to improve steatohepatitis and insulin resistance. Gut 70 (11), 2183–2195. 10.1136/gutjnl-2020-321774 33257471 PMC8515101

[B45] Sutti S. Jindal A. Locatelli I. Vacchiano M. Gigliotti L. Bozzola C. (2014). Adaptive immune responses triggered by oxidative stress contribute to hepatic inflammation in NASH. Hepatology 59 (3), 886–897. 10.1002/hep.26749 24115128

[B46] Trico D. Biancalana E. Solini A. (2021). Protein and amino acids in nonalcoholic fatty liver disease. Curr. Opin. Clin. Nutr. Metab. Care 24 (1), 96–101. 10.1097/MCO.0000000000000706 33060460

[B47] Tsuchida T. Lee Y. A. Fujiwara N. Ybanez M. Allen B. Martins S. (2018). A simple diet- and chemical-induced murine NASH model with rapid progression of steatohepatitis, fibrosis and liver cancer. J. Hepatol. 69 (2), 385–395. 10.1016/j.jhep.2018.03.011 29572095 PMC6054570

[B48] Wang L. Jiang Y. Yu Q. Xiao C. Sun J. Weng L. (2023). Gentiopicroside improves high-fat diet-induced NAFLD in association with modulation of host serum metabolome and gut microbiome in mice. Front. Microbiol. 14, 1145430. 10.3389/fmicb.2023.1145430 37614606 PMC10443917

[B49] Wishart D. S. (2019). Metabolomics for investigating physiological and pathophysiological processes. Physiol. Rev. 99 (4), 1819–1875. 10.1152/physrev.00035.2018 31434538

[B50] Xiao H. Sun X. Lin Z. Yang Y. Zhang M. Xu Z. (2022). Gentiopicroside targets PAQR3 to activate the PI3K/AKT signaling pathway and ameliorate disordered glucose and lipid metabolism. Acta Pharm. Sin. B 12 (6), 2887–2904. 10.1016/j.apsb.2021.12.023 35755276 PMC9214054

[B51] Xie G. Wang L. Chen T. Zhou K. Zhang Z. Li J. (2021). A metabolite array technology for precision medicine. Anal. Chem. 93 (14), 5709–5717. 10.1021/acs.analchem.0c04686 33797874

[B52] Yan M. Xu G. (2018). Current and future perspectives of functional metabolomics in disease studies-A review. Anal. Chim. Acta 1037, 41–54. 10.1016/j.aca.2018.04.006 30292314

[B53] Zeng M. D. Fan J. G. Lu L. G. Li Y. M. Chen C. W. Wang B. Y. (2008). Guidelines for the diagnosis and treatment of nonalcoholic fatty liver diseases. J. Dig. Dis. 9 (2), 108–112. 10.1111/j.1751-2980.2008.00331.x 18419645

[B54] Zhang X. Coker O. O. Chu E. S. Fu K. Lau H. C. H. Wang Y. X. (2021b). Dietary cholesterol drives fatty liver-associated liver cancer by modulating gut microbiota and metabolites. Gut 70 (4), 761–774. 10.1136/gutjnl-2019-319664 32694178 PMC7948195

[B55] Zhang Y. Pan S. Yi S. Sun J. Wang H. (2023). Gentiopicroside ameliorates CCl(4)-induced liver injury in mice by regulating the PPAR-γ/Nrf2 and NF-κB/IκB signaling pathways. J. Int. Med. Res. 51 (10), 3000605231204501. 10.1177/03000605231204501 37802492 PMC10560445

[B56] Zhang Y. Yang X. Wang S. Song S. Yang X. (2021a). Gentiopicroside prevents alcoholic liver damage by improving mitochondrial dysfunction in the rat model. Phytother. Res. 35 (4), 2230–2251. 10.1002/ptr.6981 33300653

[B57] Zhao M. Wang Y. Li L. Liu S. Wang C. Yuan Y. (2021). Mitochondrial ROS promote mitochondrial dysfunction and inflammation in ischemic acute kidney injury by disrupting TFAM-mediated mtDNA maintenance. Theranostics 11 (4), 1845–1863. 10.7150/thno.50905 33408785 PMC7778599

[B58] Zheng Y. Fang D. Huang C. Zhao L. Gan L. Chen Y. (2021a). Gentiana scabra restrains hepatic pro-inflammatory macrophages to ameliorate non-alcoholic fatty liver disease. Front. Pharmacol. 12, 816032. 10.3389/fphar.2021.816032 35115947 PMC8803634

[B59] Zheng Y. Huang C. Zhao L. Chen Y. Liu F. (2021b). Regulation of decorin by ursolic acid protects against non-alcoholic steatohepatitis. Biomed. Pharmacother. 143, 112166. 10.1016/j.biopha.2021.112166 34560554

[B60] Zhou Y. Orešič M. Leivonen M. Gopalacharyulu P. Hyysalo J. Arola J. (2016). Noninvasive detection of nonalcoholic steatohepatitis using clinical markers and circulating levels of lipids and metabolites. Clin. Gastroenterol. Hepatol. 14 (10), 1463–1472. 10.1016/j.cgh.2016.05.046 27317851

[B61] Zou X. Z. Zhang Y. W. Pan Z. F. Hu X. P. Xu Y. N. Huang Z. J. (2022). Gentiopicroside alleviates cardiac inflammation and fibrosis in T2DM rats through targeting Smad3 phosphorylation. Phytomedicine 106, 154389. 10.1016/j.phymed.2022.154389 36037771

[B62] Zuniga J. Cancino M. Medina F. Varela P. Vargas R. Tapia G. (2011). N-3 PUFA supplementation triggers PPAR-α activation and PPAR-α/NF-κB interaction: anti-inflammatory implications in liver ischemia-reperfusion injury. PLoS One 6 (12), e28502. 10.1371/journal.pone.0028502 22174823 PMC3234278

