# Supplementary figures and images for: Gentiopicroside improves non-alcoholic steatohepatitis by activating PPARα and suppressing HIF1
Source: Front Pharmacol. 2024 Mar 7;15:1335814. doi: 10.3389/fphar.2024.1335814 (PMC10956515; doi:10.3389/fphar.2024.1335814)

a

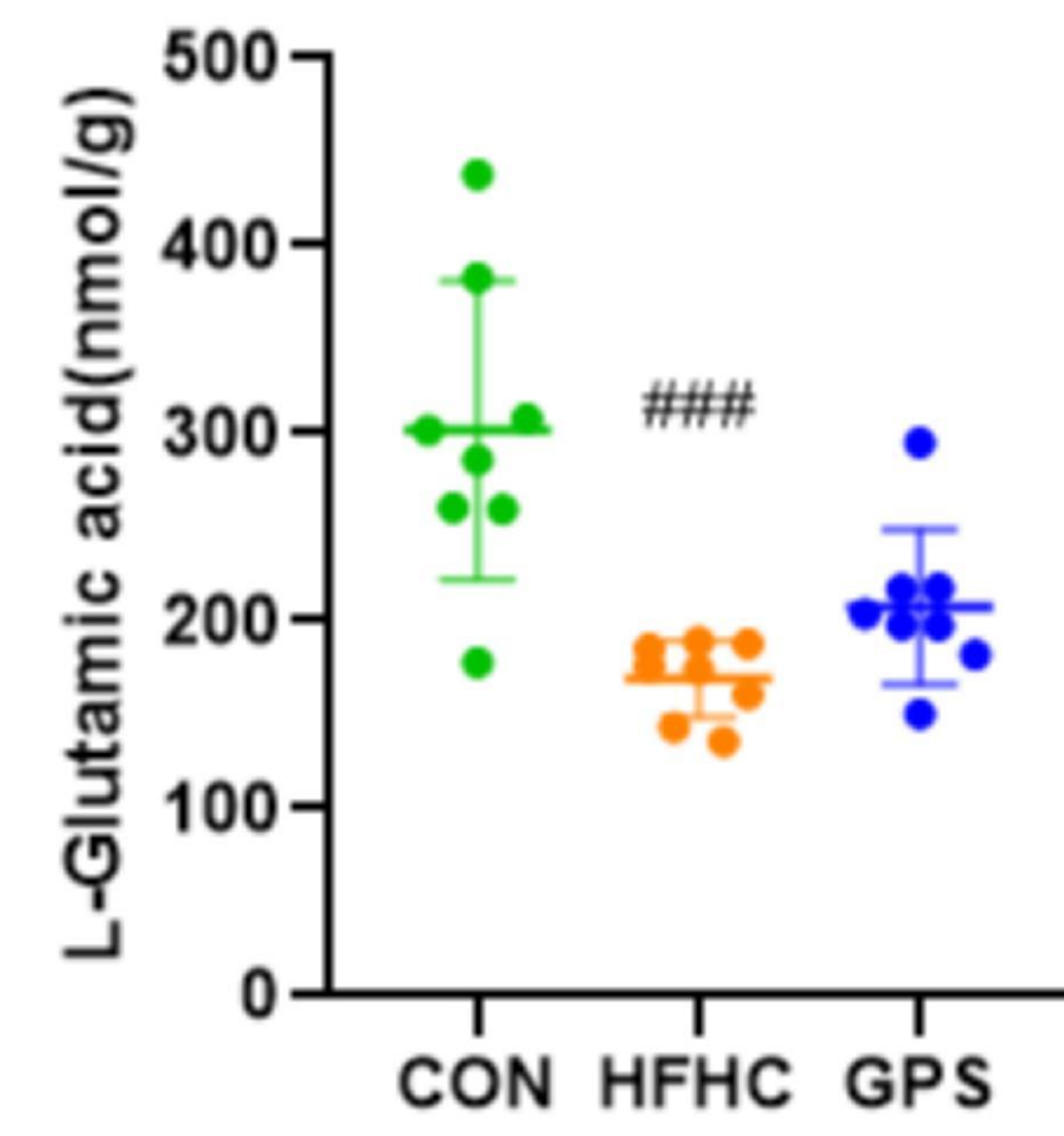

b

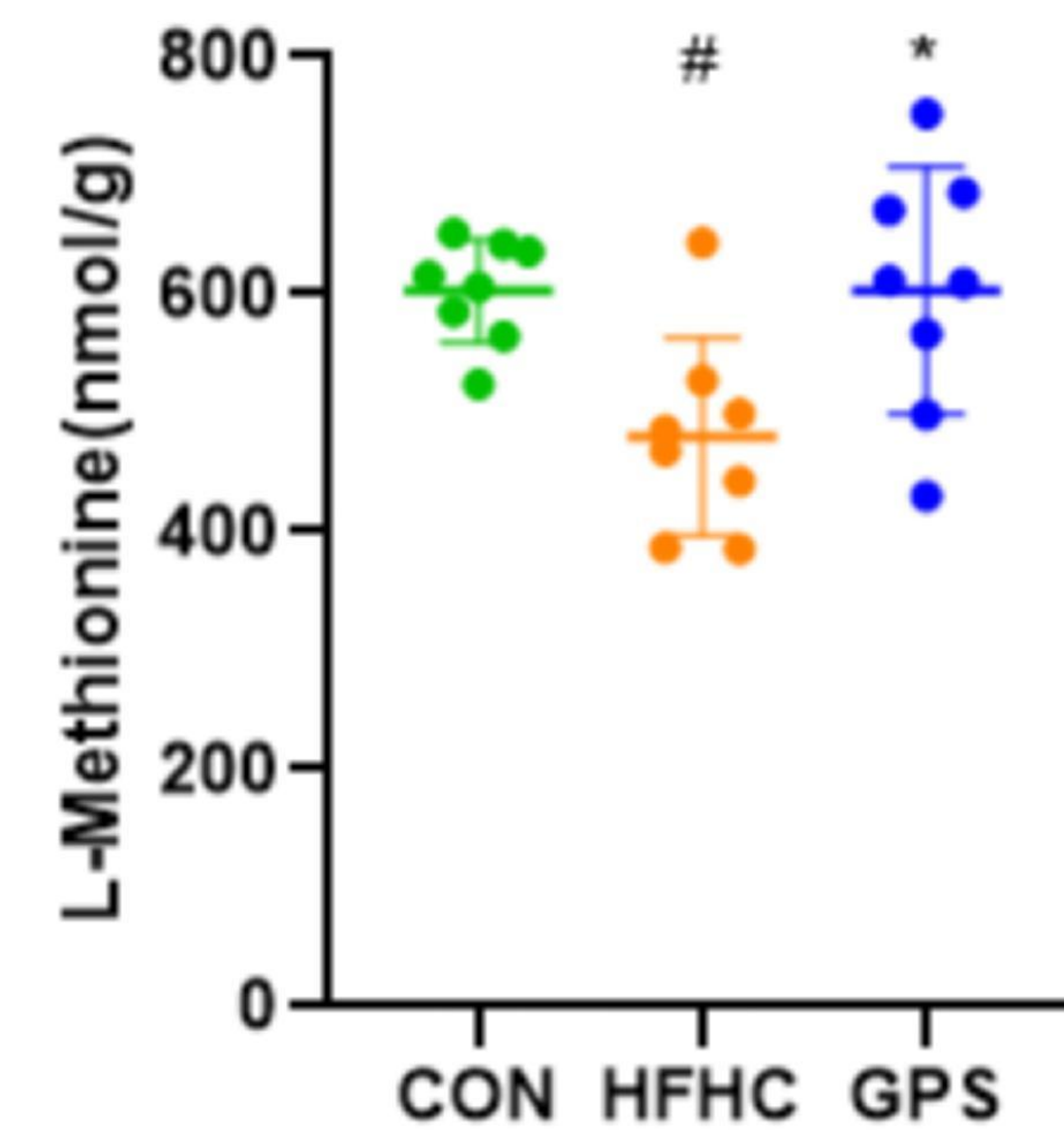

Supplement: Supplementary file 2 [file Image4.pdf]

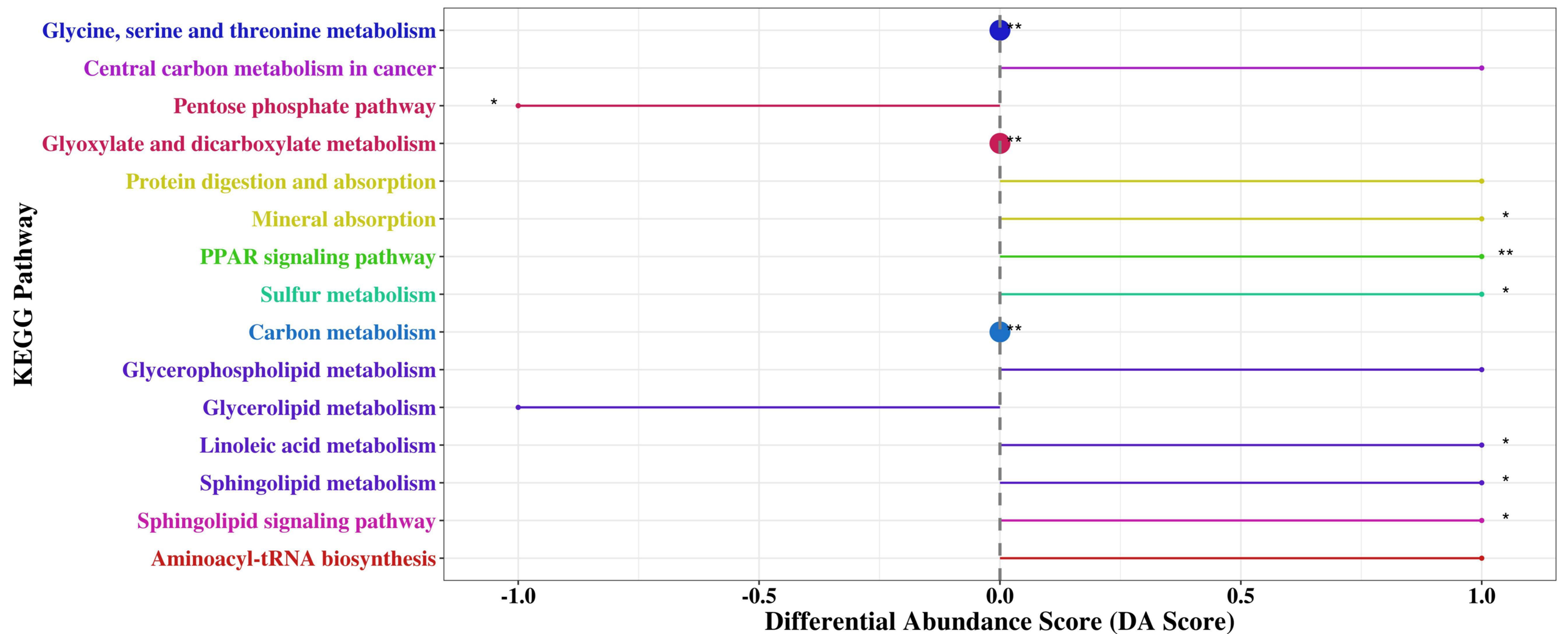

Count

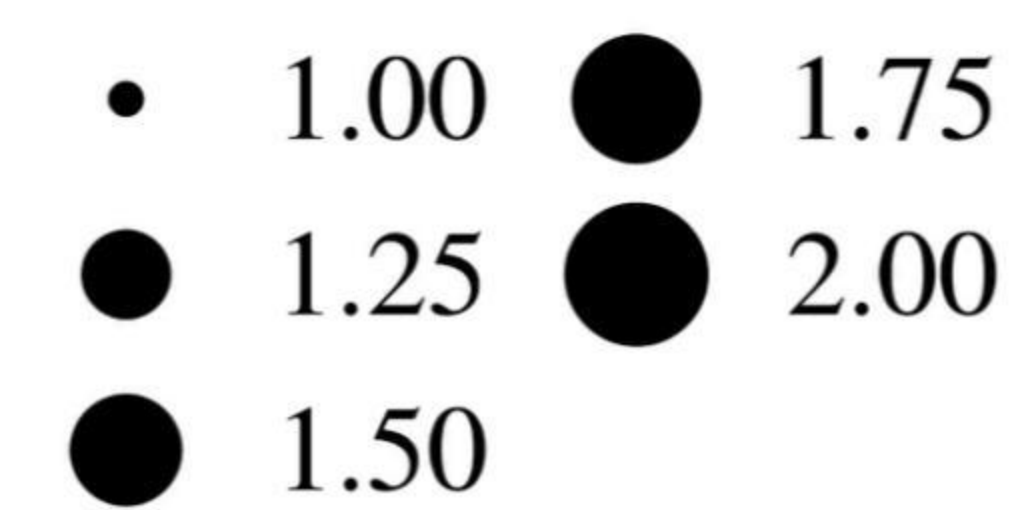

KEGG.class

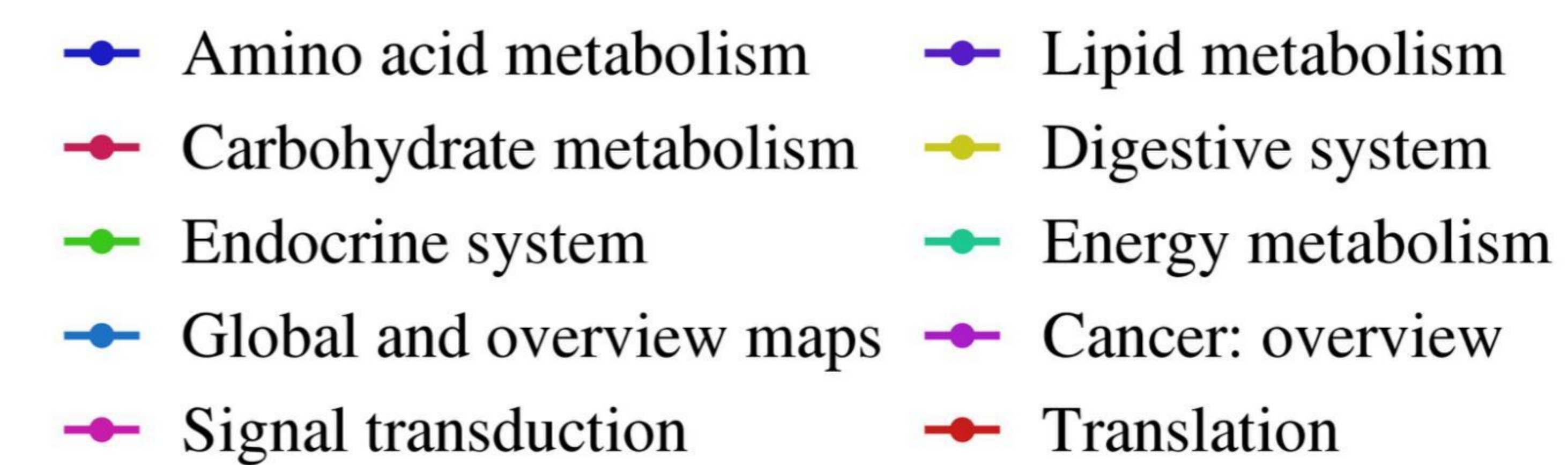

Supplement: Supplementary file 3 [file Image2.pdf]

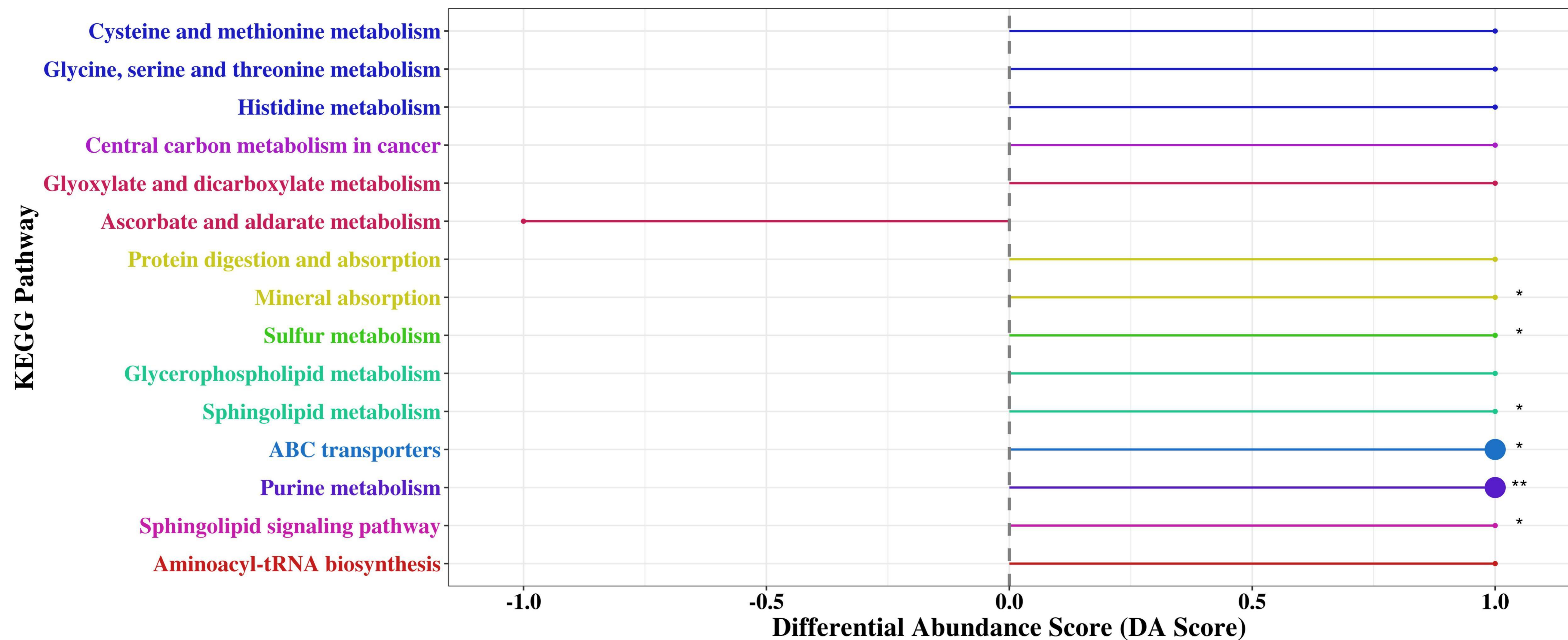

Count

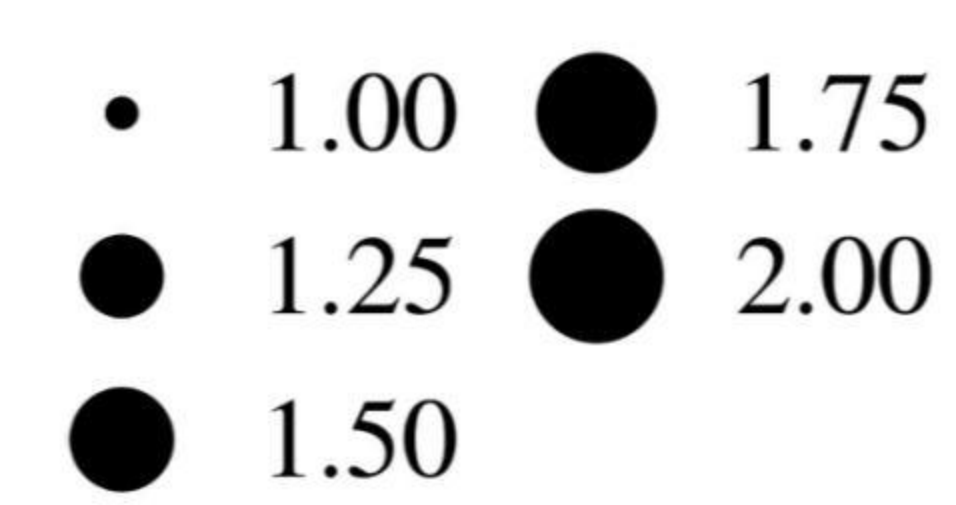

KEGG.class

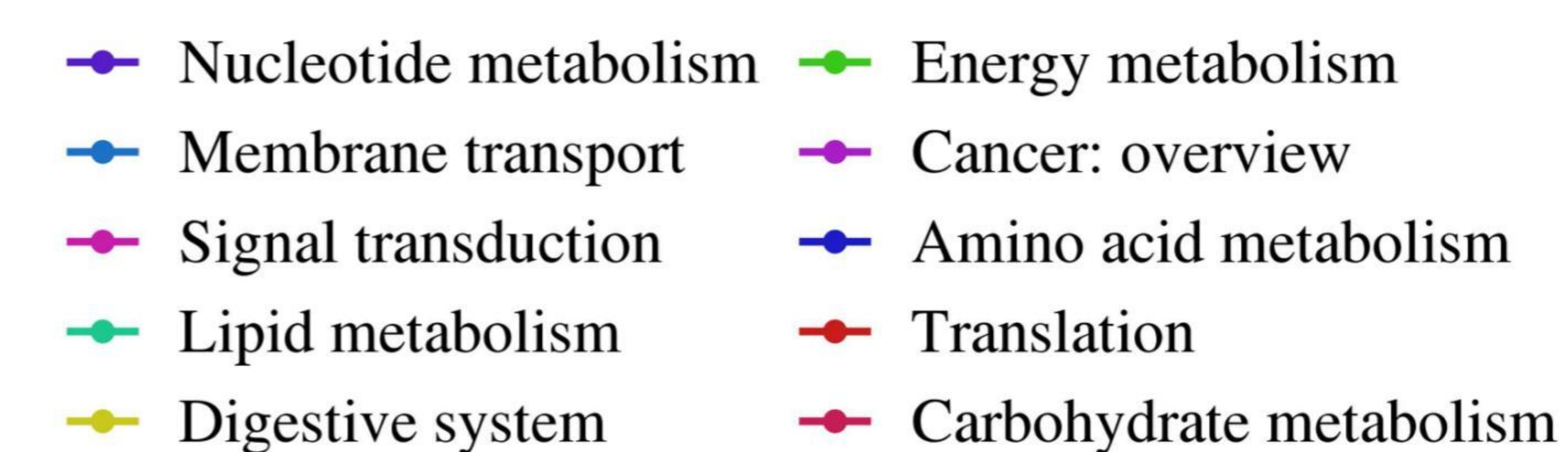

Supplement: Supplementary file 4 [file Image3.pdf]

a

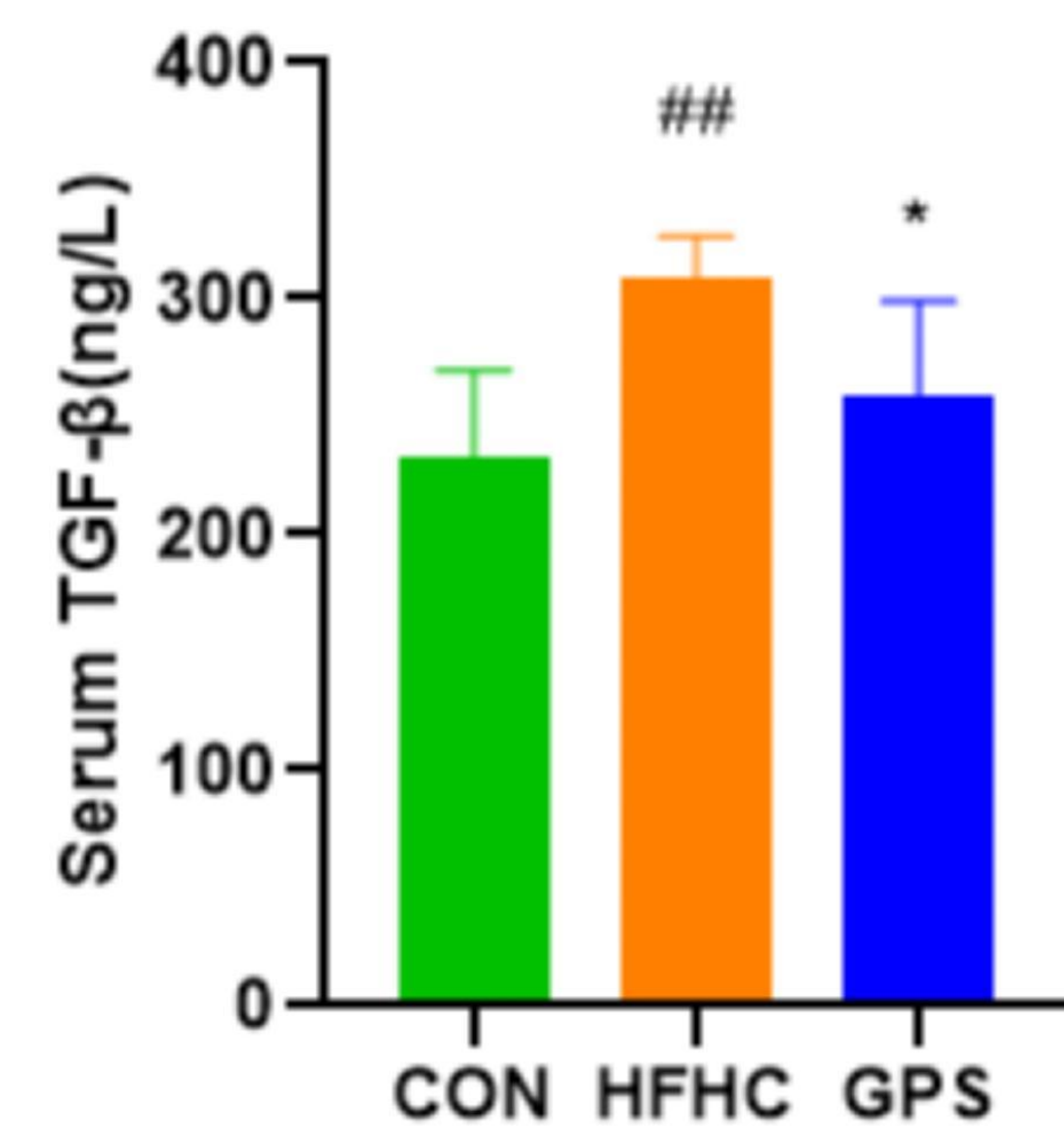

b

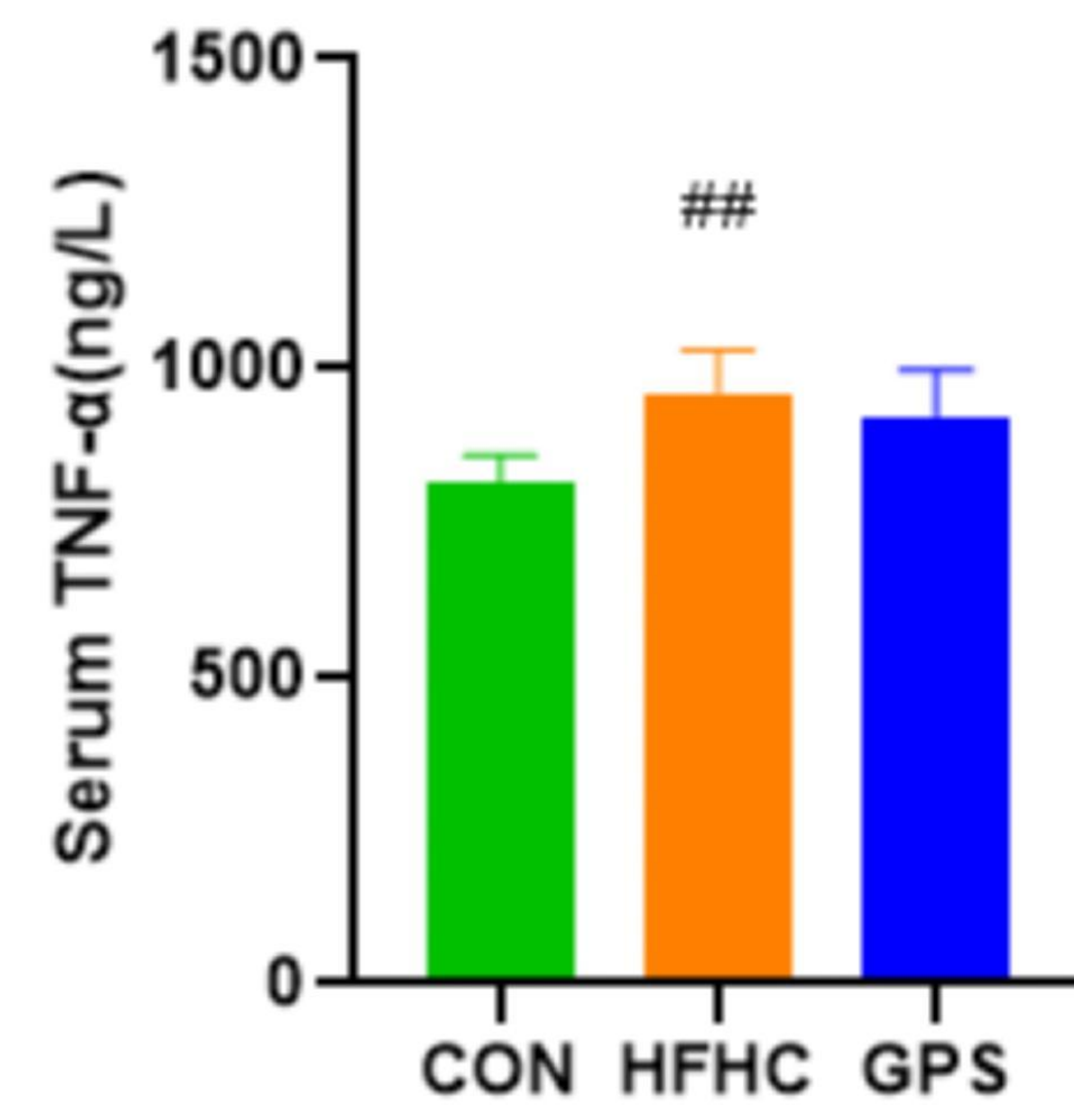

Supplement: Supplementary file 6 [file Image1.pdf]
